# Estimating uncertainty in observational studies of associations between continuous variables: example of methylmercury and neuropsychological testing in children

**DOI:** 10.1186/1742-5573-4-9

**Published:** 2007-09-26

**Authors:** Michael Goodman, Leila M Barraj, Pamela J Mink, Nicole L Britton, Janice W Yager, W Dana Flanders, Michael A Kelsh

**Affiliations:** 1 Department of Epidemiology, Rollins School of Public Health, Emory University, Atlanta, USA.; 2 Exponent, Inc., Washington, USA.; 3 Electric Power Research Institute, Palo Alto, USA.; 4 Exponent, Inc., Menlo Park, USA.

## Abstract

**Background:**

We suggest that the need to account for systematic error may explain the apparent lack of agreement among studies of maternal dietary methylmercury exposure and neuropsychological testing outcomes in children, a topic of ongoing debate.

**Methods:**

These sensitivity analyses address the possible role of systematic error on reported associations between low-level prenatal exposure to methylmercury and neuropsychological test results in two well known, but apparently conflicting cohort studies: the Faroe Islands Study (FIS) and the Seychelles Child Development Study (SCDS). We estimated the potential impact of confounding, selection bias, and information bias on reported results in these studies using the Boston Naming Test (BNT) score as the outcome variable.

**Results:**

Our findings indicate that, assuming various degrees of bias (in either direction) the corrected regression coefficients largely overlap. Thus, the reported effects in the two studies are not necessarily different from each other.

**Conclusion:**

Based on our sensitivity analysis results, it is not possible to draw definitive conclusions about the presence or absence of neurodevelopmental effects due to *in utero *methylmercury exposure at levels reported in the FIS and SCDS.

## Introduction

The potential effect of children's low-level exposure to methylmercury in the environment is a complex research issue that continues to receive considerable attention from researchers, government agencies, and the public [1]. The US Environmental Protection Agency (EPA) derived a reference dose for methylmercury in 2001, based on an analysis by the National Research Council (NRC) of the National Academy of Sciences [2]. The NRC performed benchmark dose analysis on a number of endpoints from three longitudinal prospective studies: the Seychelles Islands, the Faroe Islands, and the New Zealand studies [2]. Adverse effects were reported in the latter two studies [3-5], but not in the Seychelles study [6,7].

This lack of consistency among studies and particularly the discrepancy between the Seychelles Child Development Study (SCDS) and the Faroe Islands Studies (FIS) was noted in several previous publications [8,9]. However, most of these publications either focused on qualitative differences in the types of exposures, population characteristics and choice of endpoints between two studies [2,10], or examined the impact of non-differential measurement error in exposure assessment [11,12]. By contrast, the quantitative evaluation of systematic error in these studies does not appear to have received sufficient attention.

Current methodological literature emphasizes the importance of estimating, as opposed to merely acknowledging (or dismissing), the potential role of unaccounted systematic error in observational epidemiology [13-31] and in other fields of science [32-34]. Following these recommendations, we decided to build upon our previously published work on quantitative evaluation of potential bias in environmental epidemiologic studies [35,36] and conduct a series of sensitivity analyses to evaluate the potential impact of systematic error on the reported associations between low-level maternal dietary exposure to methylmercury and children's neuropsychological testing results in the SCDS and FIS.

We used the score of the Boston Naming Test (BNT) as the outcome variable because it seems to have received substantial attention as an endpoint of interest (NRC 2000) and because both the SCDS and the FIS have used it in their analyses. The other cohort study, conducted in New Zealand [3,5,37], did not administer the BNT.

## Methods

Our evaluation of the FIS and SCDS included two components: a qualitative review and comparison of the methods and results, and a quantitative analysis of selected sources of systematic error. The qualitative review evaluated the FIS and SCDS study methods with respect to their target population, selection of participants, exposure assessment, outcome ascertainment and data analyses. Particular attention was paid to identification of potential sources of systematic error, which were then evaluated in quantitative analyses.

The quantitative analyses presented in this article are conceptually similar to those described in our earlier publication [36] and involved calculating the impact of systematic error from three potential sources (confounding, selection bias, and information bias) on the observed relation between methylmercury exposure and a continuous neuropsychological outcome of interest.

In general terms, if a linear regression model Y = β_0 _+ β_1_X + ε represents the relation between outcome (Y) and methylmercury exposure (X), or some transformation of these (*e.g.*, Y could represent the logarithm of the dependent factor), then the least square estimate of the regression parameter β_1 _based on a sample of n observations (X_i_, Y_i_) is:

b=∑i=1nXiYi−n X¯Y¯∑i=1nXi2−nX¯2
 MathType@MTEF@5@5@+=feaafiart1ev1aaatCvAUfKttLearuWrP9MDH5MBPbIqV92AaeXatLxBI9gBamXvP5wqSXMqHnxAJn0BKvguHDwzZbqegyvzYrwyUfgarqqtubsr4rNCHbGeaGqiA8vkIkVAFgIELiFeLkFeLk=iY=Hhbbf9v8qqaqFr0xc9pk0xbba9q8WqFfeaY=biLkVcLq=JHqVepeea0=as0db9vqpepesP0xe9Fve9Fve9GapdbaqaaeGacaGaaiaabeqaamqadiabaaGcbaGaeeOyaiMaeyypa0ZaaSaaaeaadaaeWbqaaiabbIfaynaaBaaaleaacqqGPbqAaeqaaaqaaiabbMgaPjabg2da9iabbgdaXaqaaiabb6gaUbqdcqGHris5aOGaeeywaK1aaSbaaSqaaiabbMgaPbqabaGccqGHsislcqqGUbGBcuqGybawgaqeaiqbbMfazzaaraaabaWaaabCaeaacqqGybawdaqhaaWcbaGaeeyAaKgabaGaeeOmaidaaaqaaiabbMgaPjabg2da9iabbgdaXaqaaiabb6gaUbqdcqGHris5aOGaeyOeI0IaeeOBa4MafeiwaGLbaebadaahaaWcbeqaaiabbkdaYaaaaaaaaa@5FF7@

For a systematic error of certain magnitude, it is possible to estimate the corrected linear regression coefficient by accounting for this error. The impact of systematic error can also be expressed as the difference between the observed and the corrected regression coefficients (b_obs_-b). It is important to keep in mind that the sensitivity analyses presented here do not address the impact of systematic error on the epidemiologic measure of association between methyl-mercury exposure and neuropsychological testing, but rather its impact on a regression coefficient in a given study. The actual measure of association can be further affected by the model assumptions, which are beyond the scope of this paper.

As mentioned previously, the BNT score was used as the outcome variable (Y) because both the SCDS and the FIS used it in their analyses. The BNT is a 60-item test that asks the examinee to provide the name of an object depicted in black-and-white line drawings. The response that is judged to be correct and the amount of time to respond are recorded. The test can be administered with or without cues. Semantic cues, if used, are provided if no response is made within 20 seconds. If the examinee is still unable to produce the name, a phonemic cue may be provided. The total score is then the number of items correctly named spontaneously or after cues. For the Seychelles study, a score of 43 was considered normal (standard deviation of 5) [7]. Scores on the BNT are a measure of word knowledge/vocabulary, verbal learning, word retrieval, and semantic language and have been associated with reading comprehension and written comprehension [38].

The possible effect of unadjusted confounding on FIS and SCDS results was assessed by measuring the impact of potentially important covariates not considered in these studies. To estimate the impact of selection bias, we calculated the difference in BNT results that would be observed in the FIS and SCDS assuming that the distributions of exposure and BNT scores among persons omitted from these studies were different than the analogous distributions among study participants. Finally, the potential role of information bias was quantified for a given range of outcome misclassification (in either direction) differentially affecting the low exposure and the high exposure groups in each study. The derivation of the corrected linear regression estimate (b) for each specific type of systematic error was conducted as follows.

### Confounder Adjustment

Given the mathematical relationship between estimates of regression coefficients and correlation coefficients, one can use reported estimated correlation coefficients to calculate the potential impact of confounders. The correlation coefficient (*r*) for 2 variables, *Z *and *Y*, can be expressed as:

r(Z,Y)=sZsYb
 MathType@MTEF@5@5@+=feaafiart1ev1aaatCvAUfKttLearuWrP9MDH5MBPbIqV92AaeXatLxBI9gBaebbnrfifHhDYfgasaacH8akY=wiFfYdH8Gipec8Eeeu0xXdbba9frFj0=OqFfea0dXdd9vqai=hGuQ8kuc9pgc9s8qqaq=dirpe0xb9q8qiLsFr0=vr0=vr0dc8meaabaqaciaacaGaaeqabaqabeGadaaakeaacqWGYbGCcqGGOaakcqWGAbGwcqGGSaalcqWGzbqwcqGGPaqkcqGH9aqpdaWcaaqaaiabdohaZnaaBaaaleaacqWGAbGwaeqaaaGcbaGaem4Cam3aaSbaaSqaaiabdMfazbqabaaaaOGaemOyaigaaa@3B48@

where *b *is the slope of the least square regression line, and *s*_*Z *_and *s*_*Y *_are the standard deviations of Z and *Y*, respectively. Let *Y *= *b*_0 _+ *b*_*Z *_represent the fitted linear regression model relating the outcome (*Y*) to confounder *Z*. If we assume that the same regression model applies to the exposed and non-exposed populations, then:

Y¯Exp−Y¯Non−exp⁡=b(Z¯Exp−Z¯Non−exp⁡)
 MathType@MTEF@5@5@+=feaafiart1ev1aaatCvAUfKttLearuWrP9MDH5MBPbIqV92AaeXatLxBI9gBaebbnrfifHhDYfgasaacH8akY=wiFfYdH8Gipec8Eeeu0xXdbba9frFj0=OqFfea0dXdd9vqai=hGuQ8kuc9pgc9s8qqaq=dirpe0xb9q8qiLsFr0=vr0=vr0dc8meaabaqaciaacaGaaeqabaqabeGadaaakeaacuWGzbqwgaqeamaaBaaaleaacqWGfbqrcqWG4baEcqWGWbaCaeqaaOGaeyOeI0IafmywaKLbaebadaWgaaWcbaGaemOta4Kaem4Ba8MaemOBa4MaeyOeI0IagiyzauMaeiiEaGNaeiiCaahabeaakiabg2da9iabdkgaIjabcIcaOiqbdQfaAzaaraWaaSbaaSqaaiabdweafjabdIha4jabdchaWbqabaGccqGHsislcuWGAbGwgaqeamaaBaaaleaacqWGobGtcqWGVbWBcqWGUbGBcqGHsislcyGGLbqzcqGG4baEcqGGWbaCaeqaaOGaeiykaKcaaa@52C1@

which becomes

Y¯Exp−Y¯Non−exp⁡sY=r(Z,Y)×Z¯Exp−Z¯Non−exp⁡sZ
 MathType@MTEF@5@5@+=feaafiart1ev1aaatCvAUfKttLearuWrP9MDH5MBPbIqV92AaeXatLxBI9gBaebbnrfifHhDYfgasaacH8akY=wiFfYdH8Gipec8Eeeu0xXdbba9frFj0=OqFfea0dXdd9vqai=hGuQ8kuc9pgc9s8qqaq=dirpe0xb9q8qiLsFr0=vr0=vr0dc8meaabaqaciaacaGaaeqabaqabeGadaaakeaadaWcaaqaaiqbdMfazzaaraWaaSbaaSqaaiabdweafjabdIha4jabdchaWbqabaGccqGHsislcuWGzbqwgaqeamaaBaaaleaacqWGobGtcqWGVbWBcqWGUbGBcqGHsislcyGGLbqzcqGG4baEcqGGWbaCaeqaaaGcbaGaem4Cam3aaSbaaSqaaiabdMfazbqabaaaaOGaeyypa0JaemOCaiNaeiikaGIaemOwaOLaeiilaWIaemywaKLaeiykaKIaey41aq7aaSaaaeaacuWGAbGwgaqeamaaBaaaleaacqWGfbqrcqWG4baEcqWGWbaCaeqaaOGaeyOeI0IafmOwaOLbaebadaWgaaWcbaGaemOta4Kaem4Ba8MaemOBa4MaeyOeI0IagiyzauMaeiiEaGNaeiiCaahabeaaaOqaaiabdohaZnaaBaaaleaacqWGAbGwaeqaaaaaaaa@5E28@

where:

Y¯
 MathType@MTEF@5@5@+=feaafiart1ev1aaatCvAUfKttLearuWrP9MDH5MBPbIqV92AaeXatLxBI9gBaebbnrfifHhDYfgasaacH8akY=wiFfYdH8Gipec8Eeeu0xXdbba9frFj0=OqFfea0dXdd9vqai=hGuQ8kuc9pgc9s8qqaq=dirpe0xb9q8qiLsFr0=vr0=vr0dc8meaabaqaciaacaGaaeqabaqabeGadaaakeaacuWGzbqwgaqeaaaa@2DFF@_Exp _is the mean value of the outcome measure (e.g., BNT test score) among the exposed;

Y¯
 MathType@MTEF@5@5@+=feaafiart1ev1aaatCvAUfKttLearuWrP9MDH5MBPbIqV92AaeXatLxBI9gBaebbnrfifHhDYfgasaacH8akY=wiFfYdH8Gipec8Eeeu0xXdbba9frFj0=OqFfea0dXdd9vqai=hGuQ8kuc9pgc9s8qqaq=dirpe0xb9q8qiLsFr0=vr0=vr0dc8meaabaqaciaacaGaaeqabaqabeGadaaakeaacuWGzbqwgaqeaaaa@2DFF@_Non-exp _is the mean value of the outcome measure among the non-exposed;

*s*_*Y *_is the standard deviation of the outcome measure;

Z¯
 MathType@MTEF@5@5@+=feaafiart1ev1aaatCvAUfKttLearuWrP9MDH5MBPbIqV92AaeXatLxBI9gBaebbnrfifHhDYfgasaacH8akY=wiFfYdH8Gipec8Eeeu0xXdbba9frFj0=OqFfea0dXdd9vqai=hGuQ8kuc9pgc9s8qqaq=dirpe0xb9q8qiLsFr0=vr0=vr0dc8meaabaqaciaacaGaaeqabaqabeGadaaakeaacuWGAbGwgaqeaaaa@2E01@_Exp _is the mean value of the potential confounder among the exposed;

Z¯
 MathType@MTEF@5@5@+=feaafiart1ev1aaatCvAUfKttLearuWrP9MDH5MBPbIqV92AaeXatLxBI9gBaebbnrfifHhDYfgasaacH8akY=wiFfYdH8Gipec8Eeeu0xXdbba9frFj0=OqFfea0dXdd9vqai=hGuQ8kuc9pgc9s8qqaq=dirpe0xb9q8qiLsFr0=vr0=vr0dc8meaabaqaciaacaGaaeqabaqabeGadaaakeaacuWGAbGwgaqeaaaa@2E01@_Non-exp _is the mean value of the potential confounder among the non-exposed;

*s*_*Z *_is the standard deviation of the potential confounder;

and

*r*(*Z*, *Y*) is the Pearson correlation coefficient for variables Z and *Y*.

Let a multiple linear regression model Y = β_0 _+ β_1_X + β_2_Z + ε represent the relation between outcome (Y) and exposure (X) in the presence of an unaccounted confounder (Z). From the formula above, the regression parameter β_1 _corrected for unaccounted confounding can be estimated as:

bconf=r(XY)sYsX−r(XZ)sYsX×r(ZY)−r(XY)r(XZ)1−r(XZ)2
 MathType@MTEF@5@5@+=feaafiart1ev1aaatCvAUfKttLearuWrP9MDH5MBPbIqV92AaeXatLxBI9gBaebbnrfifHhDYfgasaacH8akY=wiFfYdH8Gipec8Eeeu0xXdbba9frFj0=OqFfea0dXdd9vqai=hGuQ8kuc9pgc9s8qqaq=dirpe0xb9q8qiLsFr0=vr0=vr0dc8meaabaqaciaacaGaaeqabaqabeGadaaakeaacqqGIbGydaWgaaWcbaGaee4yamMaee4Ba8MaeeOBa4MaeeOzaygabeaakiabg2da9iabbkhaYjabbIcaOiabbIfayjabbMfazjabbMcaPmaalaaabaGaee4Cam3aaSbaaSqaaiabbMfazbqabaaakeaacqqGZbWCdaWgaaWcbaGaeeiwaGfabeaaaaGccqGHsislcqqGYbGCcqqGOaakcqqGybawcqqGAbGwcqqGPaqkdaWcaaqaaiabbohaZnaaBaaaleaacqqGzbqwaeqaaaGcbaGaee4Cam3aaSbaaSqaaiabbIfaybqabaaaaOGaey41aq7aaSaaaeaacqqGYbGCcqqGOaakcqqGAbGwcqqGzbqwcqqGPaqkcqGHsislcqqGYbGCcqqGOaakcqqGybawcqqGzbqwcqqGPaqkcqqGYbGCcqqGOaakcqqGybawcqqGAbGwcqqGPaqkaeaacqqGXaqmcqGHsislcqqGYbGCcqqGOaakcqqGybawcqqGAbGwcqqGPaqkdaahaaWcbeqaaiabbkdaYaaaaaaaaa@6862@

where s_X _and s_Y _are estimates of the standard deviations of X and Y, and r(XY), r(XZ) and r(ZY) represent estimates of the correlation coefficients between X and Y, X and Z, and Z and Y, respectively. If we use formula (1) to express b_obs_, that is the estimate of the regression parameter unadjusted for the effect of confounding, then the difference (b_obs_-b_conf_) in this case represents the impact of confounding by Z on the observed linear regression coefficient.

### Selection bias

Selection bias may occur if the participants are systematically different from persons not included in the study with respect to their exposure and outcome levels. Thus, the regression slope derived from the data collected among the participants would differ from the estimate based on all eligible subjects. Let:

• n represent the total number of all eligible subjects;

• n_s _(p_s_) represent the number (proportion) of sampled subjects among the n eligible subjects;

• n_n _(p_n_) represent the number (proportion) of non-sampled subjects among the n eligible subjects;

• X¯
 MathType@MTEF@5@5@+=feaafiart1ev1aaatCvAUfKttLearuWrP9MDH5MBPbIqV92AaeXatLxBI9gBaebbnrfifHhDYfgasaacH8akY=wiFfYdH8Gipec8Eeeu0xXdbba9frFj0=OqFfea0dXdd9vqai=hGuQ8kuc9pgc9s8qqaq=dirpe0xb9q8qiLsFr0=vr0=vr0dc8meaabaqaciaacaGaaeqabaqabeGadaaakeaacuqGybawgaqeaaaa@2DFB@_s _and Y¯
 MathType@MTEF@5@5@+=feaafiart1ev1aaatCvAUfKttLearuWrP9MDH5MBPbIqV92AaeXatLxBI9gBaebbnrfifHhDYfgasaacH8akY=wiFfYdH8Gipec8Eeeu0xXdbba9frFj0=OqFfea0dXdd9vqai=hGuQ8kuc9pgc9s8qqaq=dirpe0xb9q8qiLsFr0=vr0=vr0dc8meaabaqaciaacaGaaeqabaqabeGadaaakeaacuqGzbqwgaqeaaaa@2DFD@_s _represent the estimates of the mean exposure and outcome among the sampled subjects;

• X¯
 MathType@MTEF@5@5@+=feaafiart1ev1aaatCvAUfKttLearuWrP9MDH5MBPbIqV92AaeXatLxBI9gBaebbnrfifHhDYfgasaacH8akY=wiFfYdH8Gipec8Eeeu0xXdbba9frFj0=OqFfea0dXdd9vqai=hGuQ8kuc9pgc9s8qqaq=dirpe0xb9q8qiLsFr0=vr0=vr0dc8meaabaqaciaacaGaaeqabaqabeGadaaakeaacuqGybawgaqeaaaa@2DFB@_n _and Y¯
 MathType@MTEF@5@5@+=feaafiart1ev1aaatCvAUfKttLearuWrP9MDH5MBPbIqV92AaeXatLxBI9gBaebbnrfifHhDYfgasaacH8akY=wiFfYdH8Gipec8Eeeu0xXdbba9frFj0=OqFfea0dXdd9vqai=hGuQ8kuc9pgc9s8qqaq=dirpe0xb9q8qiLsFr0=vr0=vr0dc8meaabaqaciaacaGaaeqabaqabeGadaaakeaacuqGzbqwgaqeaaaa@2DFD@_n _represent the estimates of the mean exposure and outcome among the non-sampled subjects;

• s_Xs _and s_Xn _represent the estimates of the standard deviation of the exposure levels among the sampled and non-sampled subjects, respectively (we assumed, for simplicity, that s_Xn _= s_Xs_);

• b_s _represent the estimate of the regression parameter derived using the data from the n_s _sampled subjects;

• b_n _represent the estimate of the regression parameter for the n_n _non-sampled subjects, assumed here to be a multiple of b_s_, that is b_n _= νb_s_;

• b_sel _represent the estimate of the corrected regression parameter based on all eligible subjects.

Then:

bsel=(∑XY)−1n(nX¯)(nY¯)(∑X2)−1n(nX¯)2
 MathType@MTEF@5@5@+=feaafiart1ev1aaatCvAUfKttLearuWrP9MDH5MBPbIqV92AaeXatLxBI9gBaebbnrfifHhDYfgasaacH8akY=wiFfYdH8Gipec8Eeeu0xXdbba9frFj0=OqFfea0dXdd9vqai=hGuQ8kuc9pgc9s8qqaq=dirpe0xb9q8qiLsFr0=vr0=vr0dc8meaabaqaciaacaGaaeqabaqabeGadaaakeaacqqGIbGydaWgaaWcbaGaee4CamNaeeyzauMaeeiBaWgabeaakiabg2da9maalaaabaGaeeikaGYaaabqaeaacqqGybawcqqGzbqwaSqabeqaniabggHiLdGccqqGPaqkcqGHsisldaWcaaqaaiabbgdaXaqaaiabb6gaUbaacqqGOaakcqqGUbGBcuqGybawgaqeaiabbMcaPiabbIcaOiabb6gaUjqbbMfazzaaraGaeeykaKcabaGaeeikaGYaaabqaeaacqqGybawdaahaaWcbeqaaiabbkdaYaaaaeqabeqdcqGHris5aOGaeeykaKIaeyOeI0YaaSaaaeaacqqGXaqmaeaacqqGUbGBaaGaeeikaGIaeeOBa4MafeiwaGLbaebacqqGPaqkdaahaaWcbeqaaiabbkdaYaaaaaaaaa@5482@, can be re-expressed as a function of the sums of squares, cross-products and means corresponding to the sampled and non-sampled subjects:

bsel=(∑XsYs+∑XnYn)−1n(nsX¯s+nnX¯n)(nsY¯s+nnY¯n)(∑Xs2+∑Xn2)−1n(nsX¯s+nnX¯n)2,
 MathType@MTEF@5@5@+=feaafiart1ev1aaatCvAUfKttLearuWrP9MDH5MBPbIqV92AaeXatLxBI9gBamXvP5wqSXMqHnxAJn0BKvguHDwzZbqegyvzYrwyUfgarqqtubsr4rNCHbGeaGqiA8vkIkVAFgIELiFeLkFeLk=iY=Hhbbf9v8qqaqFr0xc9pk0xbba9q8WqFfeaY=biLkVcLq=JHqVepeea0=as0db9vqpepesP0xe9Fve9Fve9GapdbaqaaeGacaGaaiaabeqaamqadiabaaGcbaGaeeOyai2aaSbaaSqaaiabbohaZjabbwgaLjabbYgaSbqabaGccqGH9aqpdaWcaaqaaiabbIcaOmaaqaeabaGaeeiwaG1aaSbaaSqaaiabbohaZbqabaGccqqGzbqwdaWgaaWcbaGaee4CamhabeaakiabgUcaRmaaqaeabaGaeeiwaG1aaSbaaSqaaiabb6gaUbqabaGccqqGzbqwdaWgaaWcbaGaeeOBa4gabeaaaeqabeqdcqGHris5aaWcbeqab0GaeyyeIuoakiabbMcaPiabgkHiTmaalaaabaGaeeymaedabaGaeeOBa4gaaiabbIcaOiabb6gaUnaaBaaaleaacqqGZbWCaeqaaOGafeiwaGLbaebadaWgaaWcbaGaee4CamhabeaakiabgUcaRiabb6gaUnaaBaaaleaacqqGUbGBaeqaaOGafeiwaGLbaebadaWgaaWcbaGaeeOBa4gabeaakiabbMcaPiabbIcaOiabb6gaUnaaBaaaleaacqqGZbWCaeqaaOGafeywaKLbaebadaWgaaWcbaGaee4CamhabeaakiabgUcaRiabb6gaUnaaBaaaleaacqqGUbGBaeqaaOGafeywaKLbaebadaWgaaWcbaGaeeOBa4gabeaakiabbMcaPaqaaiabbIcaOmaaqaeabaGaeeiwaG1aa0baaSqaaiabbohaZbqaaiabbkdaYaaakiabgUcaRmaaqaeabaGaeeiwaG1aa0baaSqaaiabb6gaUbqaaiabbkdaYaaaaeqabeqdcqGHris5aaWcbeqab0GaeyyeIuoakiabbMcaPiabgkHiTmaalaaabaGaeeymaedabaGaeeOBa4gaaiabbIcaOiabb6gaUnaaBaaaleaacqqGZbWCaeqaaOGafeiwaGLbaebadaWgaaWcbaGaee4CamhabeaakiabgUcaRiabb6gaUnaaBaaaleaacqqGUbGBaeqaaOGafeiwaGLbaebadaWgaaWcbaGaeeOBa4gabeaakiabbMcaPmaaCaaaleqabaGaeeOmaidaaaaakiabcYcaSaaa@97B9@

where the estimates of ∑X_s_Y_s _and ∑X_s_^2 ^corresponding to the sampled subjects are easily derivable by substituting the estimates of n_s_, b_s_, sXs
 MathType@MTEF@5@5@+=feaafiart1ev1aaatCvAUfKttLearuWrP9MDH5MBPbIqV92AaeXatLxBI9gBaebbnrfifHhDYfgasaacH8akY=wiFfYdH8Gipec8Eeeu0xXdbba9frFj0=OqFfea0dXdd9vqai=hGuQ8kuc9pgc9s8qqaq=dirpe0xb9q8qiLsFr0=vr0=vr0dc8meaabaqaciaacaGaaeqabaqabeGadaaakeaacqqGZbWCdaWgaaWcbaGaeeiwaG1aaSbaaWqaaiabbohaZbqabaaaleqaaaaa@3121@, X¯
 MathType@MTEF@5@5@+=feaafiart1ev1aaatCvAUfKttLearuWrP9MDH5MBPbIqV92AaeXatLxBI9gBaebbnrfifHhDYfgasaacH8akY=wiFfYdH8Gipec8Eeeu0xXdbba9frFj0=OqFfea0dXdd9vqai=hGuQ8kuc9pgc9s8qqaq=dirpe0xb9q8qiLsFr0=vr0=vr0dc8meaabaqaciaacaGaaeqabaqabeGadaaakeaacuqGybawgaqeaaaa@2DFB@_s _and Y¯
 MathType@MTEF@5@5@+=feaafiart1ev1aaatCvAUfKttLearuWrP9MDH5MBPbIqV92AaeXatLxBI9gBaebbnrfifHhDYfgasaacH8akY=wiFfYdH8Gipec8Eeeu0xXdbba9frFj0=OqFfea0dXdd9vqai=hGuQ8kuc9pgc9s8qqaq=dirpe0xb9q8qiLsFr0=vr0=vr0dc8meaabaqaciaacaGaaeqabaqabeGadaaakeaacuqGzbqwgaqeaaaa@2DFD@_s _available for the sampled subjects in standard computational formulas for the variance and linear regression parameter, to give:

∑XsYs=bs(ns−1)sXs2+nsX¯sY¯s,and∑Xs2=(ns−1)sXs2+nsX¯s2.
 MathType@MTEF@5@5@+=feaafiart1ev1aaatCvAUfKttLearuWrP9MDH5MBPbIqV92AaeXatLxBI9gBamXvP5wqSXMqHnxAJn0BKvguHDwzZbqegyvzYrwyUfgarqqtubsr4rNCHbGeaGqiA8vkIkVAFgIELiFeLkFeLk=iY=Hhbbf9v8qqaqFr0xc9pk0xbba9q8WqFfeaY=biLkVcLq=JHqVepeea0=as0db9vqpepesP0xe9Fve9Fve9GapdbaqaaeGacaGaaiaabeqaamqadiabaaGcbaqbaeaabiqaaaqaauaabeqabiaaaeaadaaeabqaaiabbIfaynaaBaaaleaacqqGZbWCaeqaaOGaeeywaK1aaSbaaSqaaiabbohaZbqabaaabeqab0GaeyyeIuoakiabg2da9iabbkgaInaaBaaaleaacqqGZbWCaeqaaOGaeeikaGIaeeOBa42aaSbaaSqaaiabbohaZbqabaGccqGHsislcqqGXaqmcqqGPaqkcqqGZbWCdaqhaaWcbaGaeeiwaG1aaSbaaWqaaiabbohaZbqabaaaleaacqqGYaGmaaGccqGHRaWkcqqGUbGBdaWgaaWcbaGaee4CamhabeaakiqbbIfayzaaraWaaSbaaSqaaiabbohaZbqabaGccuqGzbqwgaqeamaaBaaaleaacqqGZbWCaeqaaOGaeiilaWcabaGaeeyyaeMaeeOBa4MaeeizaqgaaaqaamaaqaeabaGaeeiwaG1aa0baaSqaaiabbohaZbqaaiabbkdaYaaaaeqabeqdcqGHris5aOGaeyypa0JaeeikaGIaeeOBa42aaSbaaSqaaiabbohaZbqabaGccqGHsislcqqGXaqmcqqGPaqkcqqGZbWCdaqhaaWcbaGaeeiwaG1aaSbaaWqaaiabbohaZbqabaaaleaacqqGYaGmaaGccqGHRaWkcqqGUbGBdaWgaaWcbaGaee4CamhabeaakiqbbIfayzaaraWaa0baaSqaaiabbohaZbqaaiabikdaYaaakiabc6caUaaaaaa@7ED4@

Similarly, the estimates of ∑X_n_Y_n _and ∑X_n_^2 ^corresponding to the non-sampled subjects:

∑XnYn=bn(nn−1)sXn2+nnX¯nY¯nand∑Xn2=(nn−1)sXn2+nnX¯n2
 MathType@MTEF@5@5@+=feaafiart1ev1aaatCvAUfKttLearuWrP9MDH5MBPbIqV92AaeXatLxBI9gBamXvP5wqSXMqHnxAJn0BKvguHDwzZbqegyvzYrwyUfgarqqtubsr4rNCHbGeaGqiA8vkIkVAFgIELiFeLkFeLk=iY=Hhbbf9v8qqaqFr0xc9pk0xbba9q8WqFfeaY=biLkVcLq=JHqVepeea0=as0db9vqpepesP0xe9Fve9Fve9GapdbaqaaeGacaGaaiaabeqaamqadiabaaGcbaqbaeaabiqaaaqaauaabeqabiaaaeaadaaeabqaaiabbIfaynaaBaaaleaacqqGUbGBaeqaaOGaeeywaK1aaSbaaSqaaiabb6gaUbqabaaabeqab0GaeyyeIuoakiabg2da9iabbkgaInaaBaaaleaacqqGUbGBaeqaaOGaeeikaGIaeeOBa42aaSbaaSqaaiabb6gaUbqabaGccqGHsislcqqGXaqmcqqGPaqkcqqGZbWCdaqhaaWcbaGaeeiwaG1aaSbaaWqaaiabb6gaUbqabaaaleaacqqGYaGmaaGccqGHRaWkcqqGUbGBdaWgaaWcbaGaeeOBa4gabeaakiqbbIfayzaaraWaaSbaaSqaaiabb6gaUbqabaGccuqGzbqwgaqeamaaBaaaleaacqqGUbGBaeqaaaGcbaGaeeyyaeMaeeOBa4MaeeizaqgaaaqaamaaqaeabaGaeeiwaG1aa0baaSqaaiabb6gaUbqaaiabbkdaYaaaaeqabeqdcqGHris5aOGaeyypa0JaeeikaGIaeeOBa42aaSbaaSqaaiabb6gaUbqabaGccqGHsislcqqGXaqmcqqGPaqkcqqGZbWCdaqhaaWcbaGaeeiwaG1aaSbaaWqaaiabb6gaUbqabaaaleaacqqGYaGmaaGccqGHRaWkcqqGUbGBdaWgaaWcbaGaeeOBa4gabeaakiqbbIfayzaaraWaa0baaSqaaiabb6gaUbqaaiabikdaYaaaaaaaaa@7C84@

can be estimated by substituting the hypothetical (assumed) estimates for the non-sampled subjects.

Thus (b_obs_-b_sel_) in this case represents the impact of selection bias on the observed linear regression slope.

### Information bias

In this study we assessed the impact of one type of information bias (differential outcome misclassification), which may occur when the data about the outcome are obtained differently for subjects in different exposure categories. Thus, the reported (or "observed") outcome (Y_obs_) for a proportion of the subjects is different from the "true" outcome (Y). We assume that the absolute amount of over or underestimation in the observed outcome for a subject with exposure X is proportional to the difference between X and X¯
 MathType@MTEF@5@5@+=feaafiart1ev1aaatCvAUfKttLearuWrP9MDH5MBPbIqV92AaeXatLxBI9gBaebbnrfifHhDYfgasaacH8akY=wiFfYdH8Gipec8Eeeu0xXdbba9frFj0=OqFfea0dXdd9vqai=hGuQ8kuc9pgc9s8qqaq=dirpe0xb9q8qiLsFr0=vr0=vr0dc8meaabaqaciaacaGaaeqabaqabeGadaaakeaacuqGybawgaqeaaaa@2DFB@ (the estimate of mean exposure).

Let:

• p_1 _represent the proportion of subjects whose observed outcome is Y_obs _= Y + (X-X¯
 MathType@MTEF@5@5@+=feaafiart1ev1aaatCvAUfKttLearuWrP9MDH5MBPbIqV92AaeXatLxBI9gBaebbnrfifHhDYfgasaacH8akY=wiFfYdH8Gipec8Eeeu0xXdbba9frFj0=OqFfea0dXdd9vqai=hGuQ8kuc9pgc9s8qqaq=dirpe0xb9q8qiLsFr0=vr0=vr0dc8meaabaqaciaacaGaaeqabaqabeGadaaakeaacuqGybawgaqeaaaa@2DFB@)a_1, _where a_1 _> 0. Then, p_1 _is the proportion of subjects whose bias in their observed outcome results in a positive bias in the observed slope;

• p_2 _represent the proportion of subjects whose observed outcome is Y_obs _= Y - (X-X¯
 MathType@MTEF@5@5@+=feaafiart1ev1aaatCvAUfKttLearuWrP9MDH5MBPbIqV92AaeXatLxBI9gBaebbnrfifHhDYfgasaacH8akY=wiFfYdH8Gipec8Eeeu0xXdbba9frFj0=OqFfea0dXdd9vqai=hGuQ8kuc9pgc9s8qqaq=dirpe0xb9q8qiLsFr0=vr0=vr0dc8meaabaqaciaacaGaaeqabaqabeGadaaakeaacuqGybawgaqeaaaa@2DFB@)a_2, _where a_2 _> 0. Then, p_2 _is the proportion of subjects whose bias in their observed outcome results in a negative bias in the observed slope;

• b_obs _represent the estimate of β_1 _in the regression model defined in equation (1) above, derived using Y_obs_.

Thus, Y_true _= Y_obs _-a_1_(X-X¯
 MathType@MTEF@5@5@+=feaafiart1ev1aaatCvAUfKttLearuWrP9MDH5MBPbIqV92AaeXatLxBI9gBaebbnrfifHhDYfgasaacH8akY=wiFfYdH8Gipec8Eeeu0xXdbba9frFj0=OqFfea0dXdd9vqai=hGuQ8kuc9pgc9s8qqaq=dirpe0xb9q8qiLsFr0=vr0=vr0dc8meaabaqaciaacaGaaeqabaqabeGadaaakeaacuqGybawgaqeaaaa@2DFB@) for a subset (p_1_) of all subjects, and Y_true _= Y_obs _+a_2_(X-X¯
 MathType@MTEF@5@5@+=feaafiart1ev1aaatCvAUfKttLearuWrP9MDH5MBPbIqV92AaeXatLxBI9gBaebbnrfifHhDYfgasaacH8akY=wiFfYdH8Gipec8Eeeu0xXdbba9frFj0=OqFfea0dXdd9vqai=hGuQ8kuc9pgc9s8qqaq=dirpe0xb9q8qiLsFr0=vr0=vr0dc8meaabaqaciaacaGaaeqabaqabeGadaaakeaacuqGybawgaqeaaaa@2DFB@) for a subset (p_2_) of all subjects, while Y_true _= Y_obs _for the remaining subjects.

An estimate of the regression parameter (adjusted for information bias) b_inf _is given by:

binf=∑allXYtrue−1n∑allX∑allYtrue∑allX2−1n(∑allX)2.
 MathType@MTEF@5@5@+=feaafiart1ev1aaatCvAUfKttLearuWrP9MDH5MBPbIqV92AaeXatLxBI9gBaebbnrfifHhDYfgasaacH8akY=wiFfYdH8Gipec8Eeeu0xXdbba9frFj0=OqFfea0dXdd9vqai=hGuQ8kuc9pgc9s8qqaq=dirpe0xb9q8qiLsFr0=vr0=vr0dc8meaabaqaciaacaGaaeqabaqabeGadaaakeaacqqGIbGydaWgaaWcbaGaeeyAaKMaeeOBa4MaeeOzaygabeaakiabg2da9maalaaabaWaaabuaeaacqqGybawcqqGzbqwdaWgaaWcbaGaeeiDaqNaeeOCaiNaeeyDauNaeeyzaugabeaaaeaacqqGHbqycqqGSbaBcqqGSbaBaeqaniabggHiLdGccqGHsisldaWcaaqaaiabbgdaXaqaaiabb6gaUbaadaaeqbqaaiabbIfaybWcbaGaeeyyaeMaeeiBaWMaeeiBaWgabeqdcqGHris5aOWaaabuaeaacqqGzbqwdaWgaaWcbaGaeeiDaqNaeeOCaiNaeeyDauNaeeyzaugabeaaaeaacqqGHbqycqqGSbaBcqqGSbaBaeqaniabggHiLdaakeaadaaeqbqaaiabbIfaynaaCaaaleqabaGaeeOmaidaaaqaaiabbggaHjabbYgaSjabbYgaSbqab0GaeyyeIuoakiabgkHiTmaalaaabaGaeeymaedabaGaeeOBa4gaamaabmaabaWaaabuaeaacqqGybawaSqaaiabbggaHjabbYgaSjabbYgaSbqab0GaeyyeIuoaaOGaayjkaiaawMcaamaaCaaaleqabaGaeeOmaidaaaaakiabc6caUaaa@704C@

Substituting the expressions for Y_true _in the first term in the numerator of equation 7, we get:

∑allXYtrue=∑{p1 or p2}¯XYtrue+∑p1XYtrue+∑p2XYtrue=∑{p1 or p2}¯XYobs+∑p1X[Yobs−a1(X−X¯)]+∑p2X[Yobs+a2(X−X¯)]=∑allXYobs−a1∑p1X(X−X¯)+a2∑p2X(X−X¯),
 MathType@MTEF@5@5@+=feaafiart1ev1aaatCvAUfKttLearuWrP9MDH5MBPbIqV92AaeXatLxBI9gBaebbnrfifHhDYfgasaacH8akY=wiFfYdH8Gipec8Eeeu0xXdbba9frFj0=OqFfea0dXdd9vqai=hGuQ8kuc9pgc9s8qqaq=dirpe0xb9q8qiLsFr0=vr0=vr0dc8meaabaqaciaacaGaaeqabaqabeGadaaakeaafaqadeWabaaabaWaaabuaeaacqqGybawcqqGzbqwdaWgaaWcbaGaeeiDaqNaeeOCaiNaeeyDauNaeeyzaugabeaaaeaacqqGHbqycqqGSbaBcqqGSbaBaeqaniabggHiLdGccqGH9aqpdaaeqbqaaiabbIfayjabbMfaznaaBaaaleaacqqG0baDcqqGYbGCcqqG1bqDcqqGLbqzaeqaaaqaamaanaaabaGaei4EaSNaeeiCaa3aaSbaaWqaaiabbgdaXaqabaWccqqGGaaicqqGVbWBcqqGYbGCcqqGGaaicqqGWbaCdaWgaaadbaGaeeOmaidabeaaliabc2ha9baaaeqaniabggHiLdGccqGHRaWkdaaeqbqaaiabbIfayjabbMfaznaaBaaaleaacqqG0baDcqqGYbGCcqqG1bqDcqqGLbqzaeqaaOGaey4kaScaleaacqqGWbaCdaWgaaadbaGaeeymaedabeaaaSqab0GaeyyeIuoakmaaqafabaGaeeiwaGLaeeywaK1aaSbaaSqaaiabbsha0jabbkhaYjabbwha1jabbwgaLbqabaaabaGaeeiCaa3aaSbaaWqaaiabbkdaYaqabaaaleqaniabggHiLdaakeaacqGH9aqpdaaeqbqaaiabbIfayjabbMfaznaaBaaaleaacqqGVbWBcqqGIbGycqqGZbWCaeqaaaqaamaanaaabaGaei4EaSNaeeiCaa3aaSbaaWqaaiabbgdaXaqabaWccqqGGaaicqqGVbWBcqqGYbGCcqqGGaaicqqGWbaCdaWgaaadbaGaeeOmaidabeaaliabc2ha9baaaeqaniabggHiLdGccqGHRaWkdaaeqbqaaiabbIfayjabbUfaBjabbMfaznaaBaaaleaacqqGVbWBcqqGIbGycqqGZbWCaeqaaOGaeyOeI0Iaeeyyae2aaSbaaSqaaiabbgdaXaqabaGccqqGOaakcqqGybawiiaacqWFsisldaqdaaqaaiabbIfaybaacqqGPaqkcqGGDbqxcqGHRaWkaSqaaiabbchaWnaaBaaameaacqqGXaqmaeqaaaWcbeqdcqGHris5aOWaaabuaeaacqqGybawcqqGBbWwcqqGzbqwdaWgaaWcbaGaee4Ba8MaeeOyaiMaee4CamhabeaakiabgUcaRiabbggaHnaaBaaaleaacqqGYaGmaeqaaOGaeeikaGIaeeiwaGLae8NeI0Yaa0aaaeaacqqGybawaaGaeeykaKIaeiyxa0faleaacqqGWbaCdaWgaaadbaGaeeOmaidabeaaaSqab0GaeyyeIuoaaOqaaiabg2da9maaqafabaGaeeiwaGLaeeywaK1aaSbaaSqaaiabb+gaVjabbkgaIjabbohaZbqabaaabaGaemyyaeMaemiBaWMaemiBaWgabeqdcqGHris5aOGaeyOeI0Iaemyyae2aaSbaaSqaaiabigdaXaqabaGcdaaeqbqaaiabbIfayjabbIcaOiabbIfayjab=jHiTmaanaaabaGaeeiwaGfaaiabbMcaPiabgUcaRaWcbaGaeeiCaa3aaSbaaWqaaiabbgdaXaqabaaaleqaniabggHiLdGccqWGHbqydaWgaaWcbaGaeGOmaidabeaakmaaqafabaGaeeiwaGLaeeikaGIaeeiwaGLae8NeI0Yaa0aaaeaacqqGybawaaGaeeykaKcaleaacqqGWbaCdaWgaaadbaGaeeOmaidabeaaaSqab0GaeyyeIuoakiabcYcaSaaaaaa@DF0E@

where:

∑alldenotes the sum over all observations
 MathType@MTEF@5@5@+=feaafiart1ev1aaatCvAUfKttLearuWrP9MDH5MBPbIqV92AaeXatLxBI9gBaebbnrfifHhDYfgasaacH8akY=wiFfYdH8Gipec8Eeeu0xXdbba9frFj0=OqFfea0dXdd9vqai=hGuQ8kuc9pgc9s8qqaq=dirpe0xb9q8qiLsFr0=vr0=vr0dc8meaabaqaciaacaGaaeqabaqabeGadaaakeaadaaeqbqaaiabbsgaKjabbwgaLjabb6gaUjabb+gaVjabbsha0jabbwgaLjabbohaZjabbccaGiabbsha0jabbIgaOjabbwgaLjabbccaGiabbohaZjabbwha1jabb2gaTjabbccaGiabb+gaVjabbAha2jabbwgaLjabbkhaYjabbccaGiabbggaHjabbYgaSjabbYgaSjabbccaGiabb+gaVjabbkgaIjabbohaZjabbwgaLjabbkhaYjabbAha2jabbggaHjabbsha0jabbMgaPjabb+gaVjabb6gaUjabbohaZbWcbaGaeeyyaeMaeeiBaWMaeeiBaWgabeqdcqGHris5aaaa@62D1@

∑p1denotes the sum over the proportion p1 of observations defined above
 MathType@MTEF@5@5@+=feaafiart1ev1aaatCvAUfKttLearuWrP9MDH5MBPbIqV92AaeXatLxBI9gBaebbnrfifHhDYfgasaacH8akY=wiFfYdH8Gipec8Eeeu0xXdbba9frFj0=OqFfea0dXdd9vqai=hGuQ8kuc9pgc9s8qqaq=dirpe0xb9q8qiLsFr0=vr0=vr0dc8meaabaqaciaacaGaaeqabaqabeGadaaakeaadaaeqbqaaiabbsgaKjabbwgaLjabb6gaUjabb+gaVjabbsha0jabbwgaLjabbohaZjabbccaGiabbsha0jabbIgaOjabbwgaLjabbccaGiabbohaZjabbwha1jabb2gaTjabbccaGiabb+gaVjabbAha2jabbwgaLjabbkhaYjabbccaGiabbsha0jabbIgaOjabbwgaLjabbccaGiabbchaWjabbkhaYjabb+gaVjabbchaWjabb+gaVjabbkhaYjabbsha0jabbMgaPjabb+gaVjabb6gaUjabbccaGiabbchaWnaaBaaaleaacqqGXaqmaeqaaOGaeeiiaaIaee4Ba8MaeeOzayMaeeiiaaIaee4Ba8MaeeOyaiMaee4CamNaeeyzauMaeeOCaiNaeeODayNaeeyyaeMaeeiDaqNaeeyAaKMaee4Ba8MaeeOBa4Maee4CamNaeeiiaaIaeeizaqMaeeyzauMaeeOzayMaeeyAaKMaeeOBa4MaeeyzauMaeeizaqMaeeiiaaIaeeyyaeMaeeOyaiMaee4Ba8MaeeODayNaeeyzaugaleaacqqGWbaCdaWgaaadbaGaeeymaedabeaaaSqab0GaeyyeIuoaaaa@888D@

∑p2denotes the sum over the proportion p2 of observations defined above
 MathType@MTEF@5@5@+=feaafiart1ev1aaatCvAUfKttLearuWrP9MDH5MBPbIqV92AaeXatLxBI9gBaebbnrfifHhDYfgasaacH8akY=wiFfYdH8Gipec8Eeeu0xXdbba9frFj0=OqFfea0dXdd9vqai=hGuQ8kuc9pgc9s8qqaq=dirpe0xb9q8qiLsFr0=vr0=vr0dc8meaabaqaciaacaGaaeqabaqabeGadaaakeaadaaeqbqaaiabbsgaKjabbwgaLjabb6gaUjabb+gaVjabbsha0jabbwgaLjabbohaZjabbccaGiabbsha0jabbIgaOjabbwgaLjabbccaGiabbohaZjabbwha1jabb2gaTjabbccaGiabb+gaVjabbAha2jabbwgaLjabbkhaYjabbccaGiabbsha0jabbIgaOjabbwgaLjabbccaGiabbchaWjabbkhaYjabb+gaVjabbchaWjabb+gaVjabbkhaYjabbsha0jabbMgaPjabb+gaVjabb6gaUjabbccaGiabbchaWnaaBaaaleaacqqGYaGmaeqaaOGaeeiiaaIaee4Ba8MaeeOzayMaeeiiaaIaee4Ba8MaeeOyaiMaee4CamNaeeyzauMaeeOCaiNaeeODayNaeeyyaeMaeeiDaqNaeeyAaKMaee4Ba8MaeeOBa4Maee4CamNaeeiiaaIaeeizaqMaeeyzauMaeeOzayMaeeyAaKMaeeOBa4MaeeyzauMaeeizaqMaeeiiaaIaeeyyaeMaeeOyaiMaee4Ba8MaeeODayNaeeyzaugaleaacqqGWbaCdaWgaaadbaGaeeOmaidabeaaaSqab0GaeyyeIuoaaaa@8891@

∑{p1 or p2}¯denotes the sum over all observations except the proportions p1 and p2 observations defined above.
 MathType@MTEF@5@5@+=feaafiart1ev1aaatCvAUfKttLearuWrP9MDH5MBPbIqV92AaeXatLxBI9gBaebbnrfifHhDYfgasaacH8akY=wiFfYdH8Gipec8Eeeu0xXdbba9frFj0=OqFfea0dXdd9vqai=hGuQ8kuc9pgc9s8qqaq=dirpe0xb9q8qiLsFr0=vr0=vr0dc8meaabaqaciaacaGaaeqabaqabeGadaaakeaadaaeqbqaaiabbsgaKjabbwgaLjabb6gaUjabb+gaVjabbsha0jabbwgaLjabbohaZjabbccaGiabbsha0jabbIgaOjabbwgaLjabbccaGiabbohaZjabbwha1jabb2gaTjabbccaGiabb+gaVjabbAha2jabbwgaLjabbkhaYjabbccaGiabbggaHjabbYgaSjabbYgaSjabbccaGiabb+gaVjabbkgaIjabbohaZjabbwgaLjabbkhaYjabbAha2jabbggaHjabbsha0jabbMgaPjabb+gaVjabb6gaUjabbohaZjabbccaGiabbwgaLjabbIha4jabbogaJjabbwgaLjabbchaWjabbsha0jabbccaGiabbsha0jabbIgaOjabbwgaLjabbccaGiabbchaWjabbkhaYjabb+gaVjabbchaWjabb+gaVjabbkhaYjabbsha0jabbMgaPjabb+gaVjabb6gaUjabbohaZjabbccaGiabbchaWnaaBaaaleaacqqGXaqmaeqaaOGaeeiiaaIaeeyyaeMaeeOBa4MaeeizaqMaeeiiaaIaeeiCaa3aaSbaaSqaaiabbkdaYaqabaGccqqGGaaicqqGVbWBcqqGIbGycqqGZbWCcqqGLbqzcqqGYbGCcqqG2bGDcqqGHbqycqqG0baDcqqGPbqAcqqGVbWBcqqGUbGBcqqGZbWCcqqGGaaicqqGKbazcqqGLbqzcqqGMbGzcqqGPbqAcqqGUbGBcqqGLbqzcqqGKbazcqqGGaaicqqGHbqycqqGIbGycqqGVbWBcqqG2bGDcqqGLbqzcqqGUaGlaSqaamaanaaabaGaei4EaSNaeeiCaa3aaSbaaWqaaiabbgdaXaqabaWccqqGGaaicqqGVbWBcqqGYbGCcqqGGaaicqqGWbaCdaWgaaadbaGaeeOmaidabeaaliabc2ha9baaaeqaniabggHiLdaaaa@B892@

Similarly, substituting the expressions for Y_true _in the second term in the numerator of equation 7, we get:

1n∑allX∑allYtrue=1n∑allX{∑{p1 or p2}¯Yobs+∑p1[Yobs−a1(X−X¯) ]+∑p2[Yobs+a2(X−X¯)]}=1n∑allX{∑allYobs−a1∑p1(X−X¯)+a2∑p2(X−X¯)}, or:1n∑allX∑allYtrue=1n∑allX∑allYobs−a11n∑allX∑p1(X−X¯)+a21n∑allX∑p2(X−X¯)
 MathType@MTEF@5@5@+=feaafiart1ev1aaatCvAUfKttLearuWrP9MDH5MBPbIqV92AaeXatLxBI9gBaebbnrfifHhDYfgasaacH8akY=wiFfYdH8Gipec8Eeeu0xXdbba9frFj0=OqFfea0dXdd9vqai=hGuQ8kuc9pgc9s8qqaq=dirpe0xb9q8qiLsFr0=vr0=vr0dc8meaabaqaciaacaGaaeqabaqabeGadaaakeaafaqadeWabaaabaWaaSaaaeaacqqGXaqmaeaacqqGUbGBaaWaaabuaeaacqqGybawaSqaaiabbggaHjabbYgaSjabbYgaSbqab0GaeyyeIuoakmaaqafabaGaeeywaK1aaSbaaSqaaiabbsha0jabbkhaYjabbwha1jabbwgaLbqabaaabaGaeeyyaeMaeeiBaWMaeeiBaWgabeqdcqGHris5aOGaeyypa0ZaaSaaaeaacqaIXaqmaeaacqWGUbGBaaWaaabuaeaacqWGybawaSqaaiabdggaHjabdYgaSjabdYgaSbqab0GaeyyeIuoakmaacmaabaWaaabuaeaacqqGzbqwdaWgaaWcbaGaee4Ba8MaeeOyaiMaee4CamhabeaaaeaadaqdaaqaaiabcUha7jabbchaWnaaBaaameaacqqGXaqmaeqaaSGaeeiiaaIaee4Ba8MaeeOCaiNaeeiiaaIaeeiCaa3aaSbaaWqaaiabbkdaYaqabaWccqGG9bqFaaaabeqdcqGHris5aOGaey4kaSYaaabuaeaacqqGBbWwcqqGzbqwdaWgaaWcbaGaee4Ba8MaeeOyaiMaee4CamhabeaakiabgkHiTiabbggaHnaaBaaaleaacqqGXaqmaeqaaOGaeeikaGIaeeiwaGfccaGae8NeI0Yaa0aaaeaacqqGybawaaGaeeykaKIaeeiiaaIaeiyxa0Laey4kaScaleaacqqGWbaCdaWgaaadbaGaeeymaedabeaaaSqab0GaeyyeIuoakmaaqafabaGaee4waSLaeeywaK1aaSbaaSqaaiabb+gaVjabbkgaIjabbohaZbqabaGccqGHRaWkcqqGHbqydaWgaaWcbaGaeeOmaidabeaakiabbIcaOiabbIfayjab=jHiTmaanaaabaGaeeiwaGfaaiabbMcaPiabc2faDbWcbaGaeeiCaa3aaSbaaWqaaiabbkdaYaqabaaaleqaniabggHiLdaakiaawUhacaGL9baaaeaacqGH9aqpdaWcaaqaaiabigdaXaqaaiabd6gaUbaadaaeqbqaaiabdIfaybWcbaGaemyyaeMaemiBaWMaemiBaWgabeqdcqGHris5aOWaaiWaaeaadaaeqbqaaiabbMfaznaaBaaaleaacqqGVbWBcqqGIbGycqqGZbWCaeqaaaqaaiabdggaHjabdYgaSjabdYgaSbqab0GaeyyeIuoakiabgkHiTiabdggaHnaaBaaaleaacqaIXaqmaeqaaOWaaabuaeaacqqGOaakcqqGybawcqWFsisldaqdaaqaaiabbIfaybaacqqGPaqkcqGHRaWkcqWGHbqydaWgaaWcbaGaeGOmaidabeaaaeaacqqGWbaCdaWgaaadbaGaeeymaedabeaaaSqab0GaeyyeIuoakmaaqafabaGaeeikaGIaeeiwaGLae8NeI0Yaa0aaaeaacqqGybawaaGaeeykaKcaleaacqqGWbaCdaWgaaadbaGaeeOmaidabeaaaSqab0GaeyyeIuoaaOGaay5Eaiaaw2haaiabcYcaSKaaGjabbccaGOGaee4Ba8MaeeOCaiNaeiOoaOdabaWaaSaaaeaacqqGXaqmaeaacqqGUbGBaaWaaabuaeaacqqGybawaSqaaiabbggaHjabbYgaSjabbYgaSbqab0GaeyyeIuoakmaaqafabaGaeeywaK1aaSbaaSqaaiabbsha0jabbkhaYjabbwha1jabbwgaLbqabaaabaGaeeyyaeMaeeiBaWMaeeiBaWgabeqdcqGHris5aOGaeyypa0ZaaSaaaeaacqaIXaqmaeaacqWGUbGBaaWaaabuaeaacqWGybawaSqaaiabdggaHjabdYgaSjabdYgaSbqab0GaeyyeIuoakmaaqafabaGaeeywaK1aaSbaaSqaaiabb+gaVjabbkgaIjabbohaZbqabaaabaGaemyyaeMaemiBaWMaemiBaWgabeqdcqGHris5aOGaeyOeI0Iaemyyae2aaSbaaSqaaiabigdaXaqabaGcdaWcaaqaaiabigdaXaqaaiabd6gaUbaadaaeqbqaaiabdIfaybWcbaGaemyyaeMaemiBaWMaemiBaWgabeqdcqGHris5aOWaaabuaeaacqqGOaakcqqGybawcqWFsisldaqdaaqaaiabbIfaybaacqqGPaqkcqGHRaWkcqWGHbqydaWgaaWcbaGaeGOmaidabeaakmaalaaabaGaeGymaedabaGaemOBa4gaamaaqafabaGaemiwaGfaleaacqWGHbqycqWGSbaBcqWGSbaBaeqaniabggHiLdaaleaacqqGWbaCdaWgaaadbaGaeeymaedabeaaaSqab0GaeyyeIuoakmaaqafabaGaeeikaGIaeeiwaGLae8NeI0Yaa0aaaeaacqqGybawaaGaeeykaKcaleaacqqGWbaCdaWgaaadbaGaeeOmaidabeaaaSqab0GaeyyeIuoaaaaaaa@2603@

Combining (8) and (9), the numerator of b_inf _becomes:

Numerator (binf⁡)=[∑allXYobs−1n∑allX∑allYobs]−a1[∑p1X(X−X¯)−1n∑p1X∑p1(X−X¯)]+a2[∑p2X(X−X¯)−1n∑p2X∑p2(X−X¯)]
 MathType@MTEF@5@5@+=feaafiart1ev1aaatCvAUfKttLearuWrP9MDH5MBPbIqV92AaeXatLxBI9gBaebbnrfifHhDYfgasaacH8akY=wiFfYdH8Gipec8Eeeu0xXdbba9frFj0=OqFfea0dXdd9vqai=hGuQ8kuc9pgc9s8qqaq=dirpe0xb9q8qiLsFr0=vr0=vr0dc8meaabaqaciaacaGaaeqabaqabeGadaaakeaafaqadeGabaaabaGaeeOta4KaeeyDauNaeeyBa0MaeeyzauMaeeOCaiNaeeyyaeMaeeiDaqNaee4Ba8MaeeOCaiNaeeiiaaIaeiikaGIaeeOyai2aaSbaaSqaaiGbcMgaPjabc6gaUjabcAgaMbqabaGccqGGPaqkcqGH9aqpdaWadaqaamaaqafabaGaeeiwaGLaeeywaK1aaSbaaSqaaiabb+gaVjabbkgaIjabbohaZbqabaGccqGHsisldaWcaaqaaiabigdaXaqaaiabd6gaUbaadaaeqbqaaiabdIfaybWcbaGaemyyaeMaemiBaWMaemiBaWgabeqdcqGHris5aOWaaabuaeaacqqGzbqwdaWgaaWcbaGaee4Ba8MaeeOyaiMaee4CamhabeaaaeaacqWGHbqycqWGSbaBcqWGSbaBaeqaniabggHiLdaaleaacqWGHbqycqWGSbaBcqWGSbaBaeqaniabggHiLdaakiaawUfacaGLDbaacqGHsislcqWGHbqydaWgaaWcbaGaeGymaedabeaakmaadmaabaWaaabuaeaacqqGybawcqqGOaakcqqGybawiiaacqWFsisldaqdaaqaaiabbIfaybaacqqGPaqkcqWFsisldaWcaaqaaiabigdaXaqaaiabd6gaUbaadaaeqbqaaiabdIfaybWcbaGaemiCaa3aaSbaaWqaaiabigdaXaqabaaaleqaniabggHiLdGcdaaeqbqaaiabbIcaOiabbIfayjab=jHiTmaanaaabaGaeeiwaGfaaiabbMcaPaWcbaGaemiCaa3aaSbaaWqaaiabbgdaXaqabaaaleqaniabggHiLdaaleaacqqGWbaCdaWgaaadbaGaeeymaedabeaaaSqab0GaeyyeIuoaaOGaay5waiaaw2faaaqaaiabgUcaRiabdggaHnaaBaaaleaacqaIYaGmaeqaaOWaamWaaeaadaaeqbqaaiabbIfayjabbIcaOiabbIfayjab=jHiTmaanaaabaGaeeiwaGfaaiabbMcaPaWcbaGaeeiCaa3aaSbaaWqaaiabbkdaYaqabaaaleqaniabggHiLdGccqGHsisldaWcaaqaaiabigdaXaqaaiabd6gaUbaadaaeqbqaaiabdIfaybWcbaGaemiCaa3aaSbaaWqaaiabikdaYaqabaaaleqaniabggHiLdGcdaaeqbqaaiabbIcaOiabbIfayjab=jHiTmaanaaabaGaeeiwaGfaaiabbMcaPaWcbaGaeeiCaa3aaSbaaWqaaiabbkdaYaqabaaaleqaniabggHiLdaakiaawUfacaGLDbaaaaaaaa@AE23@

If we assume that the exposure values (X) corresponding to the fractions p_1 _and p_2 _of subjects defined above are random subsamples of all X's, then, the second and third terms in equation (10) above become:

a1p1[∑all(X−X¯)2]
 MathType@MTEF@5@5@+=feaafiart1ev1aaatCvAUfKttLearuWrP9MDH5MBPbIqV92AaeXatLxBI9gBaebbnrfifHhDYfgasaacH8akY=wiFfYdH8Gipec8Eeeu0xXdbba9frFj0=OqFfea0dXdd9vqai=hGuQ8kuc9pgc9s8qqaq=dirpe0xb9q8qiLsFr0=vr0=vr0dc8meaabaqaciaacaGaaeqabaqabeGadaaakeaacqWGHbqydaWgaaWcbaGaeGymaedabeaakiabdchaWnaaBaaaleaacqaIXaqmaeqaaOWaamWaaeaadaaeqbqaaiabbIcaOiabbIfayHGaaiab=jHiTmaanaaabaGaeeiwaGfaaiabbMcaPmaaCaaaleqabaGaeeOmaidaaaqaaiabbggaHjabbYgaSjabbYgaSbqab0GaeyyeIuoaaOGaay5waiaaw2faaaaa@3FFE@, and a2p2[∑all(X−X¯)2]
 MathType@MTEF@5@5@+=feaafiart1ev1aaatCvAUfKttLearuWrP9MDH5MBPbIqV92AaeXatLxBI9gBaebbnrfifHhDYfgasaacH8akY=wiFfYdH8Gipec8Eeeu0xXdbba9frFj0=OqFfea0dXdd9vqai=hGuQ8kuc9pgc9s8qqaq=dirpe0xb9q8qiLsFr0=vr0=vr0dc8meaabaqaciaacaGaaeqabaqabeGadaaakeaacqWGHbqydaWgaaWcbaGaeGOmaidabeaakiabdchaWnaaBaaaleaacqaIYaGmaeqaaOWaamWaaeaadaaeqbqaaiabbIcaOiabbIfayHGaaiab=jHiTmaanaaabaGaeeiwaGfaaiabbMcaPaWcbaGaeeyyaeMaeeiBaWMaeeiBaWgabeqdcqGHris5aOWaaWbaaSqabeaacqaIYaGmaaaakiaawUfacaGLDbaaaaa@401E@, respectively. Thus, equation (7) becomes:

binf=[∑allXYobs−1n∑allX∑allYobs]−(a1p1−a2p2)[∑all(X−X¯)2]∑allX2−1n(∑allX)2
 MathType@MTEF@5@5@+=feaafiart1ev1aaatCvAUfKttLearuWrP9MDH5MBPbIqV92AaeXatLxBI9gBaebbnrfifHhDYfgasaacH8akY=wiFfYdH8Gipec8Eeeu0xXdbba9frFj0=OqFfea0dXdd9vqai=hGuQ8kuc9pgc9s8qqaq=dirpe0xb9q8qiLsFr0=vr0=vr0dc8meaabaqaciaacaGaaeqabaqabeGadaaakeaacqqGIbGydaWgaaWcbaGaeeyAaKMaeeOBa4MaeeOzaygabeaakiabg2da9maalaaabaWaamWaaeaadaaeqbqaaiabbIfayjabbMfaznaaBaaaleaacqqGVbWBcqqGIbGycqqGZbWCaeqaaOGaeyOeI0YaaSaaaeaacqaIXaqmaeaacqWGUbGBaaWaaabuaeaacqWGybawaSqaaiabdggaHjabdYgaSjabdYgaSbqab0GaeyyeIuoakmaaqafabaGaeeywaK1aaSbaaSqaaiabb+gaVjabbkgaIjabbohaZbqabaaabaGaemyyaeMaemiBaWMaemiBaWgabeqdcqGHris5aaWcbaGaemyyaeMaemiBaWMaemiBaWgabeqdcqGHris5aaGccaGLBbGaayzxaaGaeyOeI0IaeiikaGIaemyyae2aaSbaaSqaaiabigdaXaqabaGccqWGWbaCdaWgaaWcbaGaeGymaedabeaakiabgkHiTiabdggaHnaaBaaaleaacqaIYaGmaeqaaOGaemiCaa3aaSbaaSqaaiabikdaYaqabaGccqGGPaqkdaWadaqaamaaqafabaGaeeikaGIaeeiwaGfccaGae8NeI0Yaa0aaaeaacqqGybawaaGaeeykaKYaaWbaaSqabeaacqqGYaGmaaaabaGaeeyyaeMaeeiBaWMaeeiBaWgabeqdcqGHris5aaGccaGLBbGaayzxaaaabaWaaabuaeaacqqGybawdaahaaWcbeqaaiabbkdaYaaaaeaacqqGHbqycqqGSbaBcqqGSbaBaeqaniabggHiLdGccqGHsisldaWcaaqaaiabbgdaXaqaaiabb6gaUbaadaqadaqaamaaqafabaGaeeiwaGfaleaacqqGHbqycqqGSbaBcqqGSbaBaeqaniabggHiLdaakiaawIcacaGLPaaadaahaaWcbeqaaiabbkdaYaaaaaaaaa@8A5C@, which reduces to:

b_inf _= b_obs _- p_1_a_1 _+ p_2_a_2 _or b_obs _= b_inf _+ (p_1_)(a_1_) - (p_2_)(a_2_),

thus, (p_1_)(a_1_) - (p_2_)(a_2_), represents the magnitude of information bias (b_obs_-b_inf_).

### Monte Carlo simulations

To examine the aggregate uncertainty that results from a combination of random error and three types of systematic error (confounding, selection bias, and information bias), we used Monte Carlo simulations that included 50,000 randomly selected scenarios (Steenland and Greenland 2004). The observed distributions for FIS and SCDS were derived based on slope factors and corresponding confidence intervals reported in the original studies [7,39]. The input parameters for each Monte Carlo simulation for FIS and SCDS are summarized in Tables 1 and 2, respectively. When the data were not available, we assumed a uniform distribution reflecting a range of plausible scenarios. The adjusted distributions were derived by combining the observed distributions of the data with the distribution of the combined bias. As described previously [17,21], the events leading to the observed result could follow the following sequence: 1) effects of confounders generate population associations → 2) participants from a study are sampled from the underlying population in a manner that lead to selection bias → 3) the selected participants then become subject to differential outcome misclassification. As noted by Greenland, "this chronology suggests that we should correct misclassification first, then non-response, and then uncontrolled confounding" [17]. Adopting this approach, for each simulation iteration, the initial distribution of b_obs _after correcting for information bias served as the unadjusted distribution in the sensitivity analyses for selection bias, and the resulting slope distribution in turn was corrected for confounding producing the final adjusted distribution. All calculations were performed using Crystal Ball software (Standard Edition, 2000).

**Table 1:** Summary of input parameters and assumptions in the Monte Carlo simulation of the FIS results adjusted for outcome misclassification, selection bias and confounding

**Input Parameters**	**Distribution**	**Source (reference)**
***Outcome misclassification (information bias)***		

Observed exposure: mercury concentration in cord blood (mg/L),	Mean_x _= 31.99, SD_x _= 25.53	Budtz-Jorgensen et al. 2005; based on median and 99^th ^percentile in a log-normal distribution (39)
Observed outcome: Score on Boston naming test	Mean_y _= 25, SD_y _= 5.3	Mean: Grandjean et al. 1997 (4), SD from Budtz-Jorgensen et al. 2004
Observed b_1_	N (-0.019, 0.0063)	Budtz-Jorgensen et al. 2005 (39)
Observed b_0_	= 25 - 31.99 × Observed b_1_	Derived using standard linear regression formula (b_0 _= Y¯ MathType@MTEF@5@5@+=feaafiart1ev1aaatCvAUfKttLearuWrP9MDH5MBPbIqV92AaeXatLxBI9gBaebbnrfifHhDYfgasaacH8akY=wiFfYdH8Gipec8Eeeu0xXdbba9frFj0=OqFfea0dXdd9vqai=hGuQ8kuc9pgc9s8qqaq=dirpe0xb9q8qiLsFr0=vr0=vr0dc8meaabaqaciaacaGaaeqabaqabeGadaaakeaacuqGzbqwgaqeaaaa@2DFD@-b_1_X¯ MathType@MTEF@5@5@+=feaafiart1ev1aaatCvAUfKttLearuWrP9MDH5MBPbIqV92AaeXatLxBI9gBaebbnrfifHhDYfgasaacH8akY=wiFfYdH8Gipec8Eeeu0xXdbba9frFj0=OqFfea0dXdd9vqai=hGuQ8kuc9pgc9s8qqaq=dirpe0xb9q8qiLsFr0=vr0=vr0dc8meaabaqaciaacaGaaeqabaqabeGadaaakeaacuqGybawgaqeaaaa@2DFB@)
P1: proportion of exposed with a1 (negative) adjustment	U (0.1,0.3)	Hypothetical (no data available)
P2: proportion of exposed with a2 (positive) adjustment	U (0.1,0.3)	
a1: relative adjustment in outcome for proportion p1 of subjects	U (0.0,0.31)	Hypothetical (no data available), limits chosen to allow BNT score vary between 0 and 60
a2: relative adjustment in outcome for proportion p2 of subjects	U (0.0,0.48)	

***Selection bias***		

Observed exposure: mercury concentration in cord blood (mg/L),	See above	
10,000 vectors (Mean_y_, Sd_y_, b_0_, b_1_) adjusted for information bias	Output of Information Bias module	
Number of subjects included in the analysis	866	Grandjean et al. 1997; N in Boston Naming Test "no cues" (4)
Number of eligible subjects	1362	Calculated as 1022/0.75 (1022 are ~75% of all births (44)
Number of subjects excluded from the analysis	496	Derived as 1362-866
Relative difference between mean exposure of subjects not included and mean exposure of included subjects	U (-5%,5%)	Hypothetical (no data available)
Relative difference between mean outcome of subjects not included and mean outcome of included subjects	U (-10%,10%)	Hypothetical (no data available)
Slope multiplier (to get to slope of non-included subjects)	U (0,2)	Hypothetical (no data available)

***Confounding***		

10,000 vectors (Mean_x_, SD_x_, Mean_y_, SD_y_, b_0_, b_1_) adjusted for information and selection bias	Output of Selection Bias Module	
Pearson correlation between confounder (WAIS) and exposure	U (-0.5, 0.5)	Hypothetical (no data available)
Pearson correlation between confounder (WAIS) and outcome	U (0.2, 0.8)	Hypothetical (no data available)

N = normal distribution, U = uniform distribution

**Table 2:** Summary of input parameters and assumptions in the Monte Carlo simulation of the SCDS results adjusted for outcome misclassification, selection bias and confounding

**Input Parameters**	**Distribution**	**Source (reference)**
***Outcome misclassification (information bias)***		

Observed exposure: mercury concentration in maternal hair (mg/g)	Mean_x _= 6.9, SD_x _= 4.5	Myers et al., 2003 (7)
Observed outcome: Score on Boston naming test	Mean_y _= 26.5, SD_y _= 4.8	Myers et al., 2003 (7)
Observed b_1_	N (-0.012, 0.046)	Myers et al., 2003 (7)
Observed b_0_	= 26.5 - 6.9 × Observed b_1_	Derived using standard linear regression formula (b_0 _= Y¯ MathType@MTEF@5@5@+=feaafiart1ev1aaatCvAUfKttLearuWrP9MDH5MBPbIqV92AaeXatLxBI9gBaebbnrfifHhDYfgasaacH8akY=wiFfYdH8Gipec8Eeeu0xXdbba9frFj0=OqFfea0dXdd9vqai=hGuQ8kuc9pgc9s8qqaq=dirpe0xb9q8qiLsFr0=vr0=vr0dc8meaabaqaciaacaGaaeqabaqabeGadaaakeaacuqGzbqwgaqeaaaa@2DFD@-b_1_X¯ MathType@MTEF@5@5@+=feaafiart1ev1aaatCvAUfKttLearuWrP9MDH5MBPbIqV92AaeXatLxBI9gBaebbnrfifHhDYfgasaacH8akY=wiFfYdH8Gipec8Eeeu0xXdbba9frFj0=OqFfea0dXdd9vqai=hGuQ8kuc9pgc9s8qqaq=dirpe0xb9q8qiLsFr0=vr0=vr0dc8meaabaqaciaacaGaaeqabaqabeGadaaakeaacuqGybawgaqeaaaa@2DFB@)
P1: proportion of exposed with a1 (negative) adjustment	U (0.1,0.3)	Hypothetical (no data available)
P2: proportion of exposed with a2 (positive) adjustment	U (0.1,0.3)	
a1: relative adjustment in outcome for proportion p1 of subjects	U (0.0,1.95)	Hypothetical (no data available), limits chosen as to allow BNT score vary between 0 and 60
a2: relative adjustment in outcome for proportion p2 of subjects	U (0.0,1.95)	

***Selection bias***		

Observed exposure: mercury concentration in cord blood (mg/L)	See above	
10,000 vectors (Mean_y_, Sd_y_, b_0_, b_1_) adjusted for information bias	Output of Information Bias module	
Number of subjects included in the analysis	643	Myers et al. 2003 (7)
Number of eligible subjects	1480	Calculated as 740 × 2 (740 are ~50% of eligible population (7)
Number of subjects excluded from the analysis	837	Calculated as 1480 - 643
Relative difference between mean exposure of subjects not included and mean exposure of included subjects	U (-5%,5%)	Hypothetical (no data available)
Relative difference between mean outcome of subjects not included and mean outcome of included subjects	U (-10%,10%)	Hypothetical (no data available)
Slope multiplier (to get to slope of non-included subjects)	U (0,2)	Hypothetical (no data available)

***Confounding***		

10,000 vectors (Mean_x_, SD_x_, Mean_y_, SD_y_, b_0_, b_1_) adjusted for information and selection bias	Output of Selection Bias Module	
Pearson correlation between confounder (WAIS) and exposure	U (-0.5, 0.5)	Hypothetical (no data available)
Pearson correlation between confounder (WAIS) and outcome	U (0.2, 0.8)	Hypothetical (no data available)

N = normal distribution, U = uniform distribution

## Results

### Qualitative review of confounding

Despite rather lengthy lists of covariates that were considered in each study, the possibility remains of confounding due to unmeasured covariates or due to residual confounding. For example, no data were collected on nutritional factors (*e.g.*, selenium, polyunsaturated fatty acids) in either study [7]. Although the authors of the FIS considered confounding to have had minimal impact due to the homogeneity of the community under study and the limited potential for other neurotoxic exposures [4], it is possible that the results of this study were affected by lack of information on home environment, such as that measured by the Caldwell-Bradley Home Observation for Measurement of the Environment (HOME) [40,41]. HOME was administered to the Seychellois participants and was found to be associated with many neuropsychological tests including the Boston Naming Test [6,7]. Other variables that were either not measured, or measured but not considered consistently in the analyses, include factors related to the test-taking environment (*e.g.*, the child's anxiety level), which have been associated with performance on the WISC III Digit Spans subtest [41]; educational factors (*e.g.*, quality of school/teachers); paternal intelligence; parental education; exposure to other chemicals that have been associated with neurobehavioral effects (*e.g., *lead, PCBs); as well as dietary components, such as selenium and omega-3 fatty acids, which are expected to have a beneficial effect on neurodevelopment [42].

Both studies assessed caregiver (SCDS) or maternal (FIS) intelligence by the Raven's Progressive Matrices test rather than using a comprehensive test, such as the Wechsler Adult Intelligence Scale (WAIS). Raven's Progressive Matrices measures nonverbal reasoning ability and is a useful test for those who do not speak English. Its correlation with other intelligence tests ranges from 0.5–0.8 [41].

### Qualitative review of selection bias

Participants in the Faroe Islands study were recruited among 1,386 children from three hospitals in Torshavn, Klaksvik, and Suderoy between March 1, 1986 and December 31, 1987 [43]. Blood samples and questionnaire data were obtained from 1,023 infant-mother pairs, representing 75% of the eligible singleton births [4]. Reasons for non-participation were not described; however, it appears that patients born in two smaller hospitals were less likely to participate. It is also important to point out that the hospital with the lowest percent participation (33%) had the highest median blood mercury concentration [45].

Nine hundred seventeen of the 1,022 children returned for neuropsychological testing at approximately age seven [4]. Scores for the Boston Naming Test (no cues) were reported for 866 children, or 63% of the overall target population.

The 740 infant-mother pairs who remained in the cohort-for-analysis in the SCDS after exclusions represent approximately 50% of the target population [46]. The authors did not record specific reasons for non-participation, but indicate that some mothers were probably not informed of the study by the nurses in the hospital, some may have declined due to lack of sufficient information about the study or lack of interest, and some may have been afraid to participate in the study. Shamlaye *et al*. (1995) reported birth characteristics for SCDS participants and the target population and found small, non-significant differences in birth weight, gestational age, male:female ratio, and maternal age between the two groups [47]. Six hundred forty-three children completed the Boston Naming Test at age 108 months (9 years) in this study, which represents approximately 43% of the estimated target population.

### Qualitative review of information bias

Approximately half of all FIS participants underwent testing in the morning and half underwent testing in the afternoon. Most (but not all) children were examined in Torshavn. If the time of testing or the need to travel before testing were related to exposure, this could have introduced additional bias due to diurnal variation and/or fatigue. According to the Faroese transportation guide, long-distance bus service combined with the ferry services, links virtually every corner of the country. However, it appears that a trip to Torshavn may take up to several hours [48]. Some of the FIS participants were examined in local hospitals close to their homes. Although this may have alleviated the potential bias associated with travel, it may have introduced additional bias due to differences in testing environment.

The methods description does not indicate whether or not investigators administering the test were blinded with respect to the participants' exposure status. According to the study authors, the participation rate in the capital was lower and the participants' geometric mean mercury concentration was about 28% higher (~23 μg/L vs. ~18 μg/L) than that of non-participants. This may indicate that residence was related to both exposure level and the need to travel, as well as to the AM/PM testing status.

A re-analysis of the FIS data showed that, after controlling for residence (town vs. country), the linear regression slope for BNT without cues changed from -1.77 (p < 0.001) to -1.51 (p = 0.003), whereas the slope for BNT with cues changed from -1.91 (p < 0.001) to -1.60 (p = 0.001) [2]. However, this adjustment would only partially address the above problems. There may still be substantial room for residual misclassification because the analysis did not take into consideration distance from Torshavn or duration of travel.

Similar concerns, although to a lesser extent, apply to the SCDS results. The testing was performed "mostly in the morning." This does not exclude the potential impact of diurnal variation on the results; however, this impact would have been probably lower than that in the FIS, where the AM/PM testing ratio was 1:1.

All testing for SCDS was performed on Mahe. Some families apparently had to travel to the testing site. Similarly to the FIS, it is possible that children who had to travel were more tired prior to testing. However, one of the criteria for inclusion into the main study was Mahe residence and prolonged travel does not appear likely as Mahe extends 27 km north to south and 11 km east to west [49]. The SCDS authors state that none of the families and none of the investigators administering the test were aware of the participants' methylmercury exposure status.

### Quantitative analysis results

The results of the sensitivity analyses evaluating the potential impact of systematic error on the association between measures of methylmercury exposure and BNT scores are presented in Tables 3 through 5.

**Table 3:** Illustrative examples of FIS and SCDS BNT results corrected for unaccounted confounding

**Scenario**	**Confounder SD**	**Confounder Correlation with**		**Regression slope**	
		Exposure	Outcome	Observed	Corrected
**FIS**					
Scenario 1	15.0	-0.10	0.20	-0.019	-0.015
Scenario 2	15.0	0.10	0.20	-0.019	-0.023
Scenario 4	15.0	-0.50	0.20	-0.019	0.002
Scenario 3	15.0	0.50	0.20	-0.019	-0.053
Scenario 5	15.0	-0.10	0.80	-0.019	-0.002
Scenario 6	15.0	0.10	0.80	-0.019	-0.036
Scenario 7	15.0	-0.50	0.80	-0.019	0.085
Scenario 8	15.0	0.50	0.80	-0.019	-0.136

**SCDS**					
Scenario 1	15.0	-0.10	0.20	-0.012	0.01
Scenario 2	15.0	0.10	0.20	-0.012	-0.03
Scenario 3	15.0	-0.50	0.20	-0.012	0.13
Scenario 4	15.0	0.50	0.20	-0.012	-0.16
Scenario 5	15.0	-0.10	0.80	-0.012	0.07
Scenario 6	15.0	0.10	0.80	-0.012	-0.10
Scenario 7	15.0	-0.50	0.80	-0.012	0.55
Scenario 8	15.0	0.50	0.80	-0.012	-0.58

SD: standard deviation

**Table 4:** Illustrative examples of FIS and SCDS BNT results corrected for selection bias.

**Scenario**	**Shift in Exposure^a^**	**Shift in outcome^b^**	**Slope multiplier^c^**	**Regression slope**	
				Observed	Corrected
***FIS***					
Scenario 1	5%	10%	2.0	-0.019	-0.024
Scenario 2	5%	-10%	0.0	-0.019	-0.013
Scenario 3	-5%	-10%	1.5	-0.019	-0.021
Scenario 4	-5%	10%	2.0	-0.019	-0.027
Scenario 5	10%	10%	0.5	-0.019	-0.013
Scenario 6	10%	-10%	1.5	-0.019	-0.025
Scenario 7	-10%	-10%	0.0	-0.019	-0.009
Scenario 8	-10%	10%	0.5	-0.019	-0.018

***SCDS***					
Scenario 1	5%	10%	2.0	-0.012	0.008
Scenario 2	5%	-10%	0.0	-0.012	-0.016
Scenario 3	-5%	-10%	1.5	-0.012	-0.004
Scenario 4	-5%	10%	2.0	-0.012	-0.030
Scenario 5	10%	10%	0.5	-0.012	0.014
Scenario 6	10%	-10%	1.5	-0.012	-0.037
Scenario 7	-10%	-10%	0.0	-0.012	0.017
Scenario 8	-10%	10%	0.5	-0.012	-0.031

^a ^Difference (expressed as a percent change) between the mean exposure among the sampled subjects (X¯
 MathType@MTEF@5@5@+=feaafiart1ev1aaatCvAUfKttLearuWrP9MDH5MBPbIqV92AaeXatLxBI9gBaebbnrfifHhDYfgasaacH8akY=wiFfYdH8Gipec8Eeeu0xXdbba9frFj0=OqFfea0dXdd9vqai=hGuQ8kuc9pgc9s8qqaq=dirpe0xb9q8qiLsFr0=vr0=vr0dc8meaabaqaciaacaGaaeqabaqabeGadaaakeaacuqGybawgaqeaaaa@2DFB@_s_) compared to the entire target population which includes both sampled and non-sampled subjects (X¯
 MathType@MTEF@5@5@+=feaafiart1ev1aaatCvAUfKttLearuWrP9MDH5MBPbIqV92AaeXatLxBI9gBaebbnrfifHhDYfgasaacH8akY=wiFfYdH8Gipec8Eeeu0xXdbba9frFj0=OqFfea0dXdd9vqai=hGuQ8kuc9pgc9s8qqaq=dirpe0xb9q8qiLsFr0=vr0=vr0dc8meaabaqaciaacaGaaeqabaqabeGadaaakeaacuqGybawgaqeaaaa@2DFB@_s+n_) X¯s−(X¯s+n)X¯s×100%
 MathType@MTEF@5@5@+=feaafiart1ev1aaatCvAUfKttLearuWrP9MDH5MBPbIqV92AaeXatLxBI9gBaebbnrfifHhDYfgasaacH8akY=wiFfYdH8Gipec8Eeeu0xXdbba9frFj0=OqFfea0dXdd9vqai=hGuQ8kuc9pgc9s8qqaq=dirpe0xb9q8qiLsFr0=vr0=vr0dc8meaabaqaciaacaGaaeqabaqabeGadaaakeaadaWcaaqaaiqbbIfayzaaraWaaSbaaSqaaiabbohaZbqabaGccqGHsislcqGGOaakcuqGybawgaqeamaaBaaaleaacqqGZbWCcqGHRaWkcqqGUbGBaeqaaOGaeiykaKcabaGafeiwaGLbaebadaWgaaWcbaGaee4CamhabeaaaaGccqGHxdaTcqaIXaqmcqaIWaamcqaIWaamcqGGLaqjaaa@402B@

^b ^Difference (expressed as a percent change) between the mean outcome measure among the sampled subjects (Y¯
 MathType@MTEF@5@5@+=feaafiart1ev1aaatCvAUfKttLearuWrP9MDH5MBPbIqV92AaeXatLxBI9gBaebbnrfifHhDYfgasaacH8akY=wiFfYdH8Gipec8Eeeu0xXdbba9frFj0=OqFfea0dXdd9vqai=hGuQ8kuc9pgc9s8qqaq=dirpe0xb9q8qiLsFr0=vr0=vr0dc8meaabaqaciaacaGaaeqabaqabeGadaaakeaacuqGzbqwgaqeaaaa@2DFD@_s_) compared to the entire target population which includes both sampled an non sampled subjects (Y¯
 MathType@MTEF@5@5@+=feaafiart1ev1aaatCvAUfKttLearuWrP9MDH5MBPbIqV92AaeXatLxBI9gBaebbnrfifHhDYfgasaacH8akY=wiFfYdH8Gipec8Eeeu0xXdbba9frFj0=OqFfea0dXdd9vqai=hGuQ8kuc9pgc9s8qqaq=dirpe0xb9q8qiLsFr0=vr0=vr0dc8meaabaqaciaacaGaaeqabaqabeGadaaakeaacuqGzbqwgaqeaaaa@2DFD@_s+n_) Y¯s+Y¯s+nY¯s×100%
 MathType@MTEF@5@5@+=feaafiart1ev1aaatCvAUfKttLearuWrP9MDH5MBPbIqV92AaeXatLxBI9gBaebbnrfifHhDYfgasaacH8akY=wiFfYdH8Gipec8Eeeu0xXdbba9frFj0=OqFfea0dXdd9vqai=hGuQ8kuc9pgc9s8qqaq=dirpe0xb9q8qiLsFr0=vr0=vr0dc8meaabaqaciaacaGaaeqabaqabeGadaaakeaadaWcaaqaaiqbbMfazzaaraWaaSbaaSqaaiabbohaZbqabaGccqGHRaWkcuqGzbqwgaqeamaaBaaaleaacqqGZbWCcqGHRaWkcqqGUbGBaeqaaaGcbaGafeywaKLbaebadaWgaaWcbaGaee4CamhabeaaaaGccqGHxdaTcqaIXaqmcqaIWaamcqaIWaamcqGGLaqjaaa@3E74@

^c^Slope modifier (ν) was used to allow for scenarios where the regression slope based on the non-sampled subjects was different from the slope based on the sampled subjects: b_n _= νb_s_

**Table 5:** Illustrative examples of FIS and SCDS BNT results corrected for information bias.

**Scenario**	**Proportion misclassified**		**Magnitude of misclassification**		**Regression slope**	
	P_h_	P_h_	a_l_	a_2_	Observed	Corrected
**FIS**						
Scenario 1	30%	10%	0.30	0.40	-0.019	-0.069
Scenario 2	10%	30%	0.30	0.40	-0.019	0.071
Scenario 3	10%	10%	0.30	0.40	-0.019	-0.009
Scenario 4	30%	30%	0.30	0.40	-0.019	0.011
Scenario 5	30%	10%	0.10	0.20	-0.019	-0.029
Scenario 6	10%	30%	0.10	0.20	-0.019	0.031
Scenario 7	10%	10%	0.10	0.20	-0.019	-0.009
Scenario 8	30%	30%	0.10	0.20	-0.019	0.011

**SCDS**						
Scenario 1	30%	10%	0.30	0.40	-0.012	-0.062
Scenario 2	10%	30%	0.30	0.40	-0.012	0.078
Scenario 3	10%	10%	0.30	0.40	-0.012	-0.002
Scenario 4	30%	30%	0.30	0.40	-0.012	0.018
Scenario 5	30%	10%	0.10	0.20	-0.012	-0.022
Scenario 6	10%	30%	0.10	0.20	-0.012	0.038
Scenario 7	10%	10%	0.10	0.20	-0.012	-0.002
Scenario 8	30%	30%	0.10	0.20	-0.012	0.018

P_h _= proportion of exposed subjects whose observed BNT scores: Y_obs _= Y + (X-X¯
 MathType@MTEF@5@5@+=feaafiart1ev1aaatCvAUfKttLearuWrP9MDH5MBPbIqV92AaeXatLxBI9gBaebbnrfifHhDYfgasaacH8akY=wiFfYdH8Gipec8Eeeu0xXdbba9frFj0=OqFfea0dXdd9vqai=hGuQ8kuc9pgc9s8qqaq=dirpe0xb9q8qiLsFr0=vr0=vr0dc8meaabaqaciaacaGaaeqabaqabeGadaaakeaacuqGybawgaqeaaaa@2DFB@)a_1 _result in a positive shift in the slope

P'_h _= proportion of exposed subjects whose observed BNT scores: Y_obs _= Y - (X-X¯
 MathType@MTEF@5@5@+=feaafiart1ev1aaatCvAUfKttLearuWrP9MDH5MBPbIqV92AaeXatLxBI9gBaebbnrfifHhDYfgasaacH8akY=wiFfYdH8Gipec8Eeeu0xXdbba9frFj0=OqFfea0dXdd9vqai=hGuQ8kuc9pgc9s8qqaq=dirpe0xb9q8qiLsFr0=vr0=vr0dc8meaabaqaciaacaGaaeqabaqabeGadaaakeaacuqGybawgaqeaaaa@2DFB@)a_2 _result in a negative shift in the slope

a_1 _= relative magnitude of misclassification (positive)

a_2 _= relative magnitude of misclassification (negative)

When evaluating the possible role of unmeasured confounders in the FIS and SCDS analyses, we assumed that the correlation coefficient between confounder and exposure ranged from -0.5 to +0.5 and the correlation coefficient between confounder and outcome (BNT score) ranged from 0.2 to 0.8. The results are presented in Table 3. Based on these assumptions, the corrected regression coefficient for the FIS would become as extreme as -0.136 (Scenario 8), assuming a moderately positive correlation (r = 0.5) between the confounder and exposure and a strong correlation (r = 0.8) between the same confounder and the BNT results. On the other hand, a moderate negative correlation with exposure (r = -0.5) and a strong correlation (r = 0.8) with the outcome would reverse the direction of the association from b_obs _= -0.019 to b_conf _= +0.085 (Scenario 7). In the SCDS analyses, the same range of correlation coefficients would produce a corresponding range of corrected linear regression slopes between -0.58 (Scenario 8) and 0.55 (Scenario 7).

Table 4 illustrates the potential impact of selection bias on study results. Assuming that the differences between the mean exposures and outcomes of eligible persons who were excluded from the study and the mean exposures and outcomes of those who were included ranged between -10% and +10%, and regression slope among persons excluded from the study ranged between 0 and -0.038 (b_obs _× 2), the corrected slope for FIS may range between -0.027 (Scenario 4) and -0.009 (Scenario 7). The same selection bias scenarios in the SCDS would result in a change of direction from -0.012 to +0.017 (Scenario 7) or in a stronger than observed association, with a regression slope of -0.037 (Scenario 6).

The analyses of information bias demonstrated the effect on study results with a relatively small proportion of misclassified participants (*e.g., *10%) and the relatively modest magnitude of misclassification (a_1 _and a_2 _between 0.1 and 0.4). For the eight scenarios presented in Table 5, the corrected regression slopes ranged from -0.069 (Scenario 1) to 0.071 (Scenario 2) for FIS; and from -0.062 (Scenario 1) to 0.078 (Scenario 2) for SCDS.

**Figure 1:**
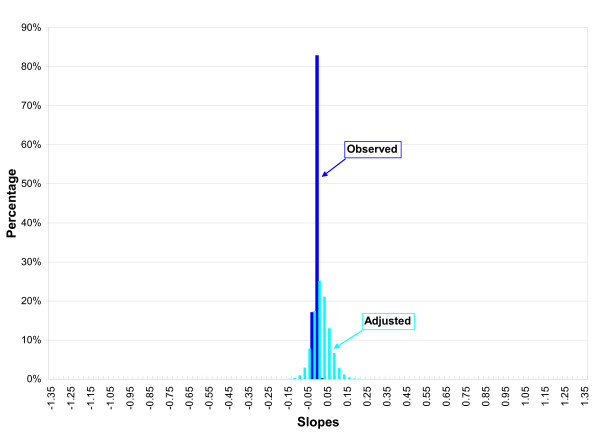
**Monte Carlo simulation of the observed and adjusted linear regression coefficients for FIS assuming various degrees of systematic error from confounding, selection bias and information bias **(unit of exposure: 1 μg/L of  cord blood).

**Figure 2:**
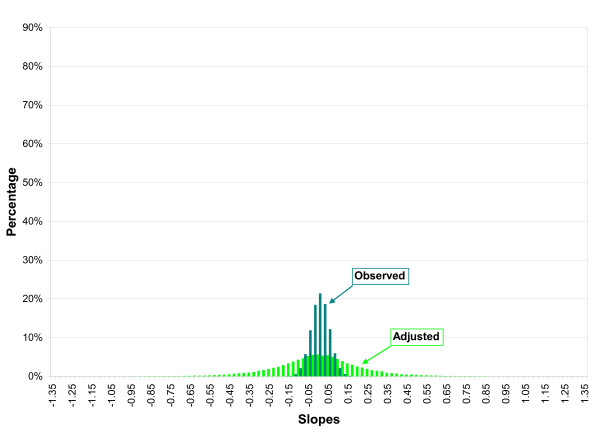
**Monte Carlo simulation of the observed and adjusted linear regression coefficients for SCDS assuming various degrees of systematic error from confounding, selection bias and information bias **(unit of exposure: 1 μg/g maternal hair)

Figures 1 and 2 illustrate the change in the distribution of the linear regression slopes assuming various degrees of combined bias (in either direction) for FIS and SCDS using the same level of random error as reported in the original studies. As shown in Figure 1, the observed distributions of FIS and SCDS results demonstrate apparently conflicting findings. However, if the FIS and SCDS study results for BNT were subject to mild-to-moderate bias from all three sources, the adjusted linear regression distributions are no longer inconsistent and the overall uncertainty makes the results of the two studies more similar.

## Discussion

A comparison of the two studies included in our analysis revealed a number of similarities. Both were prospective evaluations of neuropsychological endpoints in children whose prenatal methylmercury exposure status was ascertained at birth. Both used objective biomarker-based measures of exposure. Both conducted multivariate analyses in an attempt to separate the effects of methylmercury from other factors that influence neuropsychological function.

Yet, despite similarities, the results and conclusions of these two studies were inconsistent. For example, testing of the language function showed a statistically significant improvement with increasing methylmercury exposure among Seychellois children at about 51/2 years of age when measured by the Preschool Language Scale and no significant association at nine years of age when measured by BNT. In contrast, the Faroese study group displayed a statistically significant decline in BNT scores with increasing methylmercury exposure at the age of seven. Other discrepancies between the two sets of results were present in the domains of the visual-spatial function, memory, learning achievement, and sustained attention. Only in one domain (motor function) did both studies report statistically significant inverse associations between test scores and methylmercury exposure, but those associations were not consistent. In the SCDS, the association was for the "non-dominant" hand grooved pegboard test among males only, whereas the FIS reported the association for the "preferred" hand finger tapping.

The proposed interpretations of the observed disagreement between the two studies have been based primarily on the assumption that the differences in results have an underlying biological explanation. Recent reviews paid substantial attention to the fact that the two studies reported their main findings using different measures of methylmercury exposure: cord blood versus maternal hair [2,10]. As cord blood concentrations measure recent exposures, the National Academy of Sciences review on methylmercury toxicity suggested that the FIS results may reflect a more recent (and presumably more relevant) period of exposure [2]. Another proposed explanation is the difference in the source and rate of methylmercury exposure: daily consumption of fish in the Seychelles as opposed to episodic consumption of whale in the Faroes.

Prior to the publication of the most recent SCDS update, it appeared plausible that the differences between the two study results could also be explained by the lack of comparability in the neuropsychological test batteries. However, the last testing of the SCDS participants included many of the same tests previously used by the FIS investigators – specifically, those with significant findings – and the above explanation no longer appears likely.

Our analyses indicate that each of the potential sources of systematic error under certain conditions is capable of changing the results from significant to non-significant and vice versa. Moreover, under some scenarios even the direction of the observed associations can be reversed. Although the scenarios in our sensitivity analyses cover a wide range of assumptions, they are not entirely hypothetical. The differences in exposure levels between participants and non-participants in the FIS have been reported [4,45] and, in fact, exceed the differences assumed in our selection bias simulation. The low (just over 40%) participation rate in the SCDS also falls within the proposed scenarios. We demonstrated the potential effect of confounding by home environment and the need for a comprehensive parental IQ evaluation in our earlier publication [36]. The correlation coefficients between potential confounders and exposure are similar to those reported in the FIS. The potential misclassification due to fatigue, timing and sequencing of testing and lack of adequate blinding also finds support in the literature [38,41].

For all of the above reasons, the uncertainty around the FIS and the SCDS regression slope estimates is probably larger than is suggested by the reported 95% confidence intervals. The discrepant results of the two studies may, in fact, fall within an expected range and departures from null in either direction can be explained by a combination of random and systematic error.

The interpretation of sensitivity analyses presented here, just like the interpretation of any epidemiological analyses, requires careful consideration of caveats and underlying assumptions. Many sensitivity analyses, including ours, are limited by insufficient information (*e.g., *lack of data on the correlation between confounder and exposure) and have to rely on hypothetical distributions of the parameters of interest. When no data were available, we assumed a uniform distribution in the Monte Carlo analyses. We recognize that the uniform distribution may not accurately reflect the uncertainty since all values within the range are given equal probabilities. In the future, alternative approaches such as the use of triangular or beta distributions, which give more weight to the more "probable" values, may need to be explored. The assumptions of normal distribution and independence of various sources of bias also need to be considered and alternative analytical methods for circumstances that do not fit these assumptions may need to be developed. For example, our adjustment for unmeasured confounders does not condition on the variables for which adjustment was made. It is important to point out that adjusting for the measured covariates may reduce the residual confounding attributable to the unmeasured confounder. All of the above considerations may affect the results of sensitivity analyses; however, in the absence of sensitivity analyses, one implicitly assumes that systematic error had no effect on study results, an assumption that may be even more difficult to defend.

In summary, despite caveats, we feel that our analyses served their purpose of illustrating the proposed methodology. We conclude that sensitivity analyses serve as an important tool in understanding the sources of such disagreement as long as the underlying assumptions are clearly stated. It is important to recognize that disagreement across studies is one of the unavoidable features of observational epidemiology.

## References

[B1] Stern AH (1999). Effects of methylmercury exposure on neurodevelopment. JAMA.

[B2] NRC NRC (2000). Toxicological Effects of Methylmercury.

[B3] Crump KS (1998). Influence of prenatal mercury exposure upon scholastic and psychological test performance: Benchmark analysis of a New Zealand cohort. Risk Analysis.

[B4] Grandjean P (1997). Cognitive deficit in 7-year-old children with prenatal exposure to methylmercury. Neurotoxicol Teratol.

[B5] Kjellstrom T (1989). Physical and Mental Development of Children with Prenatal Exposure to Mercury from Fish. Stage 2: Interviews and Psychological Tests at Age 6.

[B6] Davidson PW (1998). Effects of prenatal and postnatal methylmercury exposure from fish consumption on neurodevelopment: Outcomes at 66 months of age in the Seychelles Child Development Study. JAMA.

[B7] Myers GJ (2003). Prenatal methylmercury exposure from ocean fish consumption in the Seychelles Child Development Study. Lancet.

[B8] Dourson ML, Wullenweber AE, Poirier KA (2001). Uncertainties in the reference dose for methylmercury. Neurotoxicology.

[B9] Jacobson JL (2001). Contending with contradictory data in a risk assessment context: The case of methylmercury. Neurotoxicology.

[B10] Myers GJ (2000). Twenty-seven years studying the human neurotoxicity of methylmercury exposure. Environ Res.

[B11] Budtz-Jorgensen E, Keiding N, Grandjean P, Weihe P, White RF (2003). Consequences of exposure measurement error for confounder identification in environmental epidemiology. Stat Med.

[B12] Keiding N, Budtz-Jorgensen E, Grandjean P (2003). Prenatal methylmercury exposure in the Seychelles. Lancet.

[B13] Greenland S (1996). Basic methods for sensitivity analysis of biases. Int J Epidemiol.

[B14] Greenland S, Rothman KJGS (1998). Basic methods for sensitivity analysis and external adjustment. Modern Epidemiology.

[B15] Greenland S (2001). Sensitivity analysis, Monte Carlo risk analysis, and Bayesian uncertainty assessment. Risk Anal.

[B16] Greenland S (2003). The impact of prior distributions for uncontrolled confounding and response bias: a case study of the relation of wire codes and magnetic fields to childhood leukemia. Journal of the American Statistical Association.

[B17] Greenland S (2005). Multiple-bias modeling for analysis of observational data. J R Statist Soc A.

[B18] Gustafson P (2003). Measurement Error and Misclassification in Statistics and Epidemiology.

[B19] Lash TL (2003). Semi-automated sensitivity analysis to assess systematic errors in observational data. Epidemiology.

[B20] Lash TL, Silliman RA (2000). A sensitivity analysis to separate bias due to confounding from bias due to predicting misclassification by a variable that does both. Epidemiology.

[B21] Maclure M, Schneeweiss S (2001). Causation of bias: the episcope.. Epidemiology.

[B22] Maldonado G (1998). Informal evaluation of bias may be inadequate (abstract). American Journal of Epidemiology.

[B23] Maldonado G (2003). Occupational exposure to glycol ethers and human congenital malformations. Int Arch Occup Environ Health.

[B24] Maldonado G (2005). Quantifying the impact of study imperfections on study results (abstract). American Journal of Epidemiology.

[B25] Maldonado G, Delzell E, Poole C (1999). A unified approach to conducting and interpreting occupational studies of congenital malformations (abstract). American Journal of Epidemiology.

[B26] Maldonado G, Greenland S (2002). Estimating causal effects. Int J Epidemiol.

[B27] Marais ML (1998). Correcting for omitted-variables and measurement-error bias in regression with an application to the effect of lead on IQ. J Am Stat Assoc.

[B28] Phillips CV (2003). Quantifying and reporting uncertainty from systematic errors. Epidemiology.

[B29] Phillips CV, G M (1999). Using Monte Carlo methods to quantify the multiple sources of error in studies (abstract).. American Journal of Epidemiology.

[B30] Phillips CV, LaPole LM (2003). Quantifying errors without random sampling. BMC Med Res Methodol.

[B31] Steenland K, Greenland S (2004). Monte Carlo sensitivity analysis and Bayesian analysis of smoking as an unmeasured confounder in a study of silica and lung cancer. Am J Epidemiol.

[B32] Leamer EE (1985). Sensitivity analyses would help. Am Econ Rev.

[B33] Morgan MG, Henrion M (1990). Uncertainty. A Guide to Dealing With Uncertainty in Quantitative Risk and Policy Analysis.

[B34] Vose D (2000). Risk Analysis. A Quantitative Guide.

[B35] Goodman M (2002). Evaluation of potential confounders in planning a study of occupational magnetic field exposure and female breast cancer. Epidemiology.

[B36] Mink PJ, Goodman M, Barraj LM, Imrey H, Kelsh MA, Yager J (2004). Evaluation of uncontrolled confounding in studies of environmental exposures and neurobehavioral testing in children. Epidemiology.

[B37] Kjellstrom T (1986). Physical and Mental Development of Children with Prenatal Exposure to Mercury from Fish. Stage 1: Preliminary Tests at Age 4.

[B38] Baron IS (2004). Neuropsychological Evaluation of the Child.

[B39] Budtz-Jorgensen E, Debes F, Weihe P, Grandjean P (2005). Adverse Mercury Effects in 7 Year-Old Children as Expressed as Loss in “IQ”. Final report to the EPA.

[B40] Bradley RH (1984). The relation of infants' home environments to achievement test performance in first grade:  A follow-up study. Child Dev.

[B41] Sattler JM (2001). Assessment of Children: Cognitive Applications.

[B42] Steuerwald U, Weihe P, Jorgensen PJ, Bjerve K, Brock J, Heinzow B, Budtz-Jorgensen E, Grandjean P (2000). Maternal seafood diet, methylmercury exposure, and neonatal neurologic function. J Pediatr.

[B43] Grandjean P (1992). Impact of maternal seafood diet on fetal exposure to mercury, selenium, and lead. Arch Environ Health.

[B44] Dahl R, White RF, Weihe P, Sorensen N, Letz R, Hudnell HK, Otto DA, Grandjean P (1996). Feasibility and validity of three computer-assisted neurobehavioral tests in 7-year-old children. Neurotoxicol Teratol.

[B45] Grandjean P, Weihe P (1993). Neurobehavioral effects of intrauterine mercury exposure: potential sources of bias. Environ Res.

[B46] Marsh DO (1995). The Seychelles study of fetal methylmercury exposure and child development: Introduction. Neurotoxicology.

[B47] Shamlaye CF (1995). The Seychelles child development study on neurodevelopmental outcomes in children following in utero exposure to methylmercury from a maternal fish diet: background and demographics. Neurotoxicology.

[B48] Strandfaraskip Landsins Ferdaælanin, http://www.ssl.fo. http://www.ssl.fo.

[B49] Africa Guide Seychelles http://www.africaguide.com/country/seychel. http://www.africaguide.com/country/seychel.

